# A rise-to-threshold process for a relative-value decision

**DOI:** 10.1038/s41586-023-06271-6

**Published:** 2023-07-05

**Authors:** Vikram Vijayan, Fei Wang, Kaiyu Wang, Arun Chakravorty, Atsuko Adachi, Hessameddin Akhlaghpour, Barry J. Dickson, Gaby Maimon

**Affiliations:** 1 Laboratory of Integrative Brain Function and Howard Hughes Medical Institute, The Rockefeller University, New York, NY USA; 2 Janelia Research Campus, Howard Hughes Medical Institute, Ashburn, VA USA; 3 Queensland Brain Institute, University of Queensland, St Lucia, Queensland Australia; 4 Present Address: Institute of Neuroscience, State Key Laboratory of Neuroscience, Center for Excellence in Brain Science and Intelligence Technology, Chinese Academy of Sciences, Shanghai, China; 5 Present Address: Lingang Laboratory, Shanghai Center for Brain Science and Brain-Inspired Intelligence Technology, Shanghai, China; 6 Present Address: Division of Biology and Biological Engineering, California Institute of Technology, Pasadena, CA USA; 7 Present Address: Mortimer B. Zuckerman Mind Brain Behavior Institute, Columbia University, New York, NY USA

**Keywords:** Decision, Decision

## Abstract

Whereas progress has been made in the identification of neural signals related to rapid, cued decisions^1–3^, less is known about how brains guide and terminate more ethologically relevant decisions in which an animal’s own behaviour governs the options experienced over minutes^4–6^. *Drosophila* search for many seconds to minutes for egg-laying sites with high relative value^7,8^ and have neurons, called oviDNs, whose activity fulfills necessity and sufficiency criteria for initiating the egg-deposition motor programme^9^. Here we show that oviDNs express a calcium signal that (1) dips when an egg is internally prepared (ovulated), (2) drifts up and down over seconds to minutes—in a manner influenced by the relative value of substrates—as a fly determines whether to lay an egg and (3) reaches a consistent peak level just before the abdomen bend for egg deposition. This signal is apparent in the cell bodies of oviDNs in the brain and it probably reflects a behaviourally relevant rise-to-threshold process in the ventral nerve cord, where the synaptic terminals of oviDNs are located and where their output can influence behaviour. We provide perturbational evidence that the egg-deposition motor programme is initiated once this process hits a threshold and that subthreshold variation in this process regulates the time spent considering options and, ultimately, the choice taken. Finally, we identify a small recurrent circuit that feeds into oviDNs and show that activity in each of its constituent cell types is required for laying an egg. These results argue that a rise-to-threshold process regulates a relative-value, self-paced decision and provide initial insight into the underlying circuit mechanism for building this process.

## Abstract (web summary)

The *Drosophila* egg-deposition motor programme is initiated once a rise-to-threshold process hits a threshold, and subthreshold variation in this process regulates the time spent considering options.

## Main

Egg-laying site selection is critical for the survival of a fly’s progeny^10^. As such, *Drosophila* search for a high-quality substrate for many seconds to minutes before depositing each individual egg^7,8^. Egg-laying preferences for many different substrates have been documented^10^, but how decision-related neural signals evolve in real time to guide the site selection process, and to generate these preferences, is unknown.

## A behavioural sequence for egg laying

**Fig. 1:**
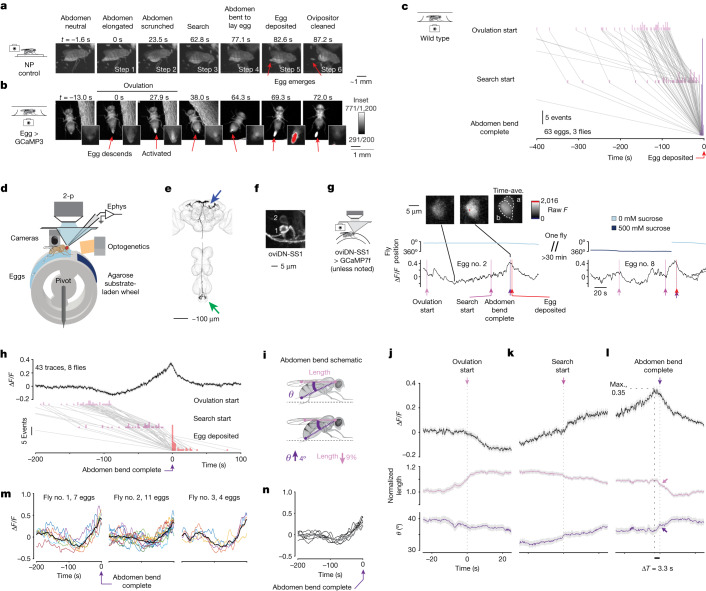
oviDN [Ca^2+^] dips during ovulation, rises for seconds to minutes and peaks immediately before the abdomen bend for egg deposition. **a**, Behavioural sequence of egg laying. **b**, Egg expressing GCaMP3 in the body. Steps correspond to **a**. Insets show close-ups, with over/undersaturated pixels in red/blue; main panels show over/undersaturated pixels in white/black. **c**, Behavioural progression. Lines connect single egg-laying sequences. **d**, Schematic of wheel. **e**, Single oviDNb traced from light microscopy images. Blue arrow indicates soma in brain, green arrow indicates outputs in the abdominal ganglion. **f**, oviDN somas on the right side of the brain labelled by oviDN-SS1. **g**, oviDN ∆*F*/*F* and behaviour during laying of two eggs by the same fly. ∆*F*/*F* is smoothed with a 2 s boxcar filter. Images are *z*-projection of selected imaging slices, with labels referring to oviDNa and oviDNb (oviDNa is partially obscured by oviDNb). **h**, Population-averaged oviDNb ∆*F*/*F* aligned to the end of the abdomen bend for egg laying. Light grey shading represents ±s.e.m. throughout; 43 imaging traces from 41 egg-laying events associated with nine cells in eight flies. The number of traces exceeds the number of egg-laying events because for two eggs we imaged oviDNb on both sides of the brain. Behavioural events shown below. **i**, Schematic of abdomen bend. *θ* denotes ‘body angle’ and length is neck–ovipositor distance. **j**–**l**, Mean oviDN ∆*F*/*F* and behaviour aligned to events in **h**: ‘ovulation start’ (**j**), ‘search start’ (**k**) and completion of abdomen bend (**l**). ‘Normalized length’ is the length given in **i** divided by its median (Methods). Shorter, thicker arrows indicate when abdomen bend for egg deposition is complete. A subsequent (stronger) bend is, presumably, for cleaning the ovipositor. **m**, oviDN ∆*F*/*F* during individual egg-laying events, smoothed with a 5 s boxcar filter. Black line, mean. **n**, Mean oviDN ∆*F*/*F* during egg laying for all seven flies that laid three or more eggs, smoothed with a 5 s boxcar filter. A single GCaMP7b fly is shown in grey. NP, Nippon Project; Ave., average; 2-p, two-photon; Ephys, electrophysiology; Max., maximum.

We took videos of gravid *Drosophila* in a small chamber with a soft substrate floor and characterized a behavioural sequence for egg laying (see Supplementary Tables 1 and 2 for genotypes and conditions in all experiments). The six-step sequence begins with the fly standing still and performing an abdomen elongation (step 1) followed by a scrunch (step 2) (Fig. 1a). The fly then increases its locomotor speed during a search period (step 3), and finally it performs an abdomen bend for egg deposition (step 4), deposits an egg (step 5) and performs a second abdomen bend (step 6), probably for cleaning the ovipositor.

**Extended Data Fig. 1:**
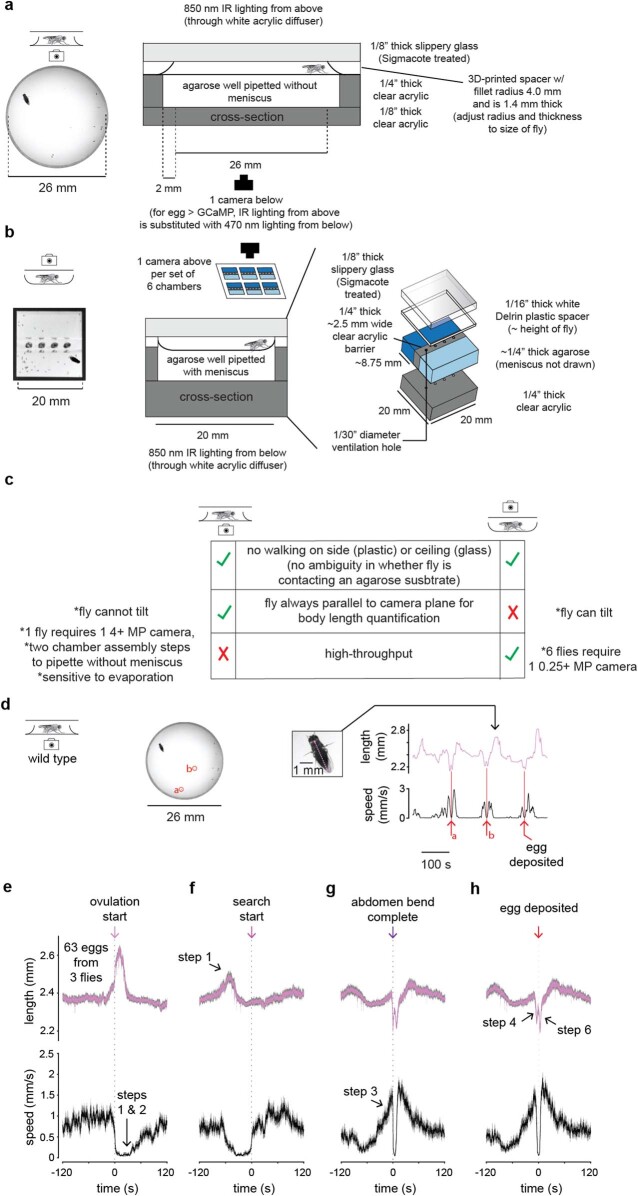
Free behavior chambers and characterization of the egg-laying behavioral sequence. **a**, Schematic of free behavior egg-laying chamber with sloped ceiling. **b**, Schematic of high-throughput free behavior egg-laying choice chamber. **c**, Comparison of the two free behavior chamber types. **d**, Length (neck to ovipositor distance) and locomotor speed over 3 consecutive egg-laying events, smoothed with a 5 s boxcar filter, for a single fly in a sloped ceiling egg-laying chamber (Supplementary Video 2). **e-h**, Mean length and locomotor speed aligned to annotated events in the egg-laying behavioral sequence. Light grey shading is ± s.e.m. Prominent features of steps from Fig. 1a are labeled. These features (e.g., the pause or increase in locomotion) were not considered when annotating the event used for alignment.

This sequence is consistent with those described previously^7,9,11–13^ and, although abdominal movements before egg laying have been noted^11–13^, it remains unclear whether any of these reflect ovulation^14^, which is the passage of an egg from an ovary to the uterus. We fluorescently imaged, through the cuticle, eggs expressing GCaMP^15^ while freely walking flies laid eggs (Extended Data Fig. 1a and Methods). By visualization of GCaMP rather than green fluorescent protein (GFP), we could determine not only when eggs moved inside the body but also when each egg was activated to start embryonic development (because activation is associated with a large [Ca^2+^] increase inside the egg^15^). We observed that an egg descends from an ovary to the uterus during abdominal elongation and that the same egg exhibits a strong increase in GCaMP fluorescence during the subsequent scrunch (Fig. 1b and Supplementary Video 1). These data demonstrate that elongation (step 1) reflects ovulation and that scrunching (step 2) reflects activation. For brevity we will refer to steps 1 and 2, combined, as ovulation in this paper.

We quantified the egg-laying behavioural sequence by annotating four of the six steps just mentioned: (1) ovulation start (when the abdomen first begins to elongate), (2) search start (when the abdomen returns to a neutral posture after ovulation), (3) ‘abdomen bend complete’ (when the abdomen shows its maximum deflection before egg deposition) and (4) egg deposition (when half of the egg is visible outside the ovipositor) (Fig. 1c, Extended Data Fig. 1d–h, Supplementary Video 2 and Methods). We observed substantial inter-egg variation in search duration—that is, the time between search start and completion of the abdomen bend for egg deposition (Fig. 1c). Because the decision to lay an egg is made within this variable time window, we sought to find a neural signal whose dynamics in this time period could illuminate the decision process.

## Neurophysiology during egg laying

**Extended Data Fig. 2:**
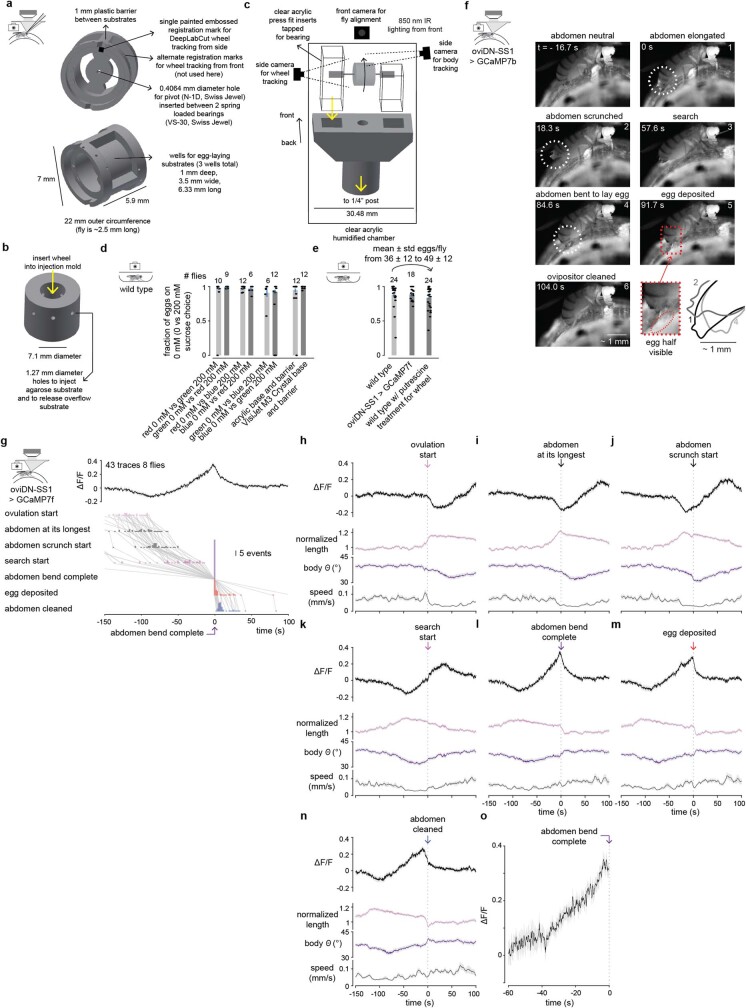
Egg-laying wheel and tethered egg-laying behavioral sequence with oviDN [Ca^2+^]. **a**, Schematic of egg-laying wheel. **b**, Schematic of agarose-injecting mold, which is used to load agarose onto the wheel with a pipette. **c**, Schematic of egg-laying wheel assembly secured in a custom humidification chamber under the microscope objective. **d**, Fraction of eggs on the lower sucrose option for control substrates: colored dye infused substrates and 3D-printer material (VisiJet M3 Crystal) bases vs. acrylic bases. Error bars represent 95% confidence intervals. Each dot is one fly. These data suggest that the dyes and plastics involved in fabricating the egg-laying wheel should not cause abnormal substrate choice behavior. **e**, Fraction of eggs on the lower sucrose option for flies expressing GCaMP7f in oviDNs and by those pre-treated for tethered wheel experiments. Error bars represent 95% confidence intervals. Each dot is one fly. These data show that the flies we used in our imaging experiments exhibit normal substrate choice during free-behavior egg laying. **f**, Behavioral sequence of tethered egg laying as in Fig. 1a. Stills from a single egg-laying event. Overlaid and zoomed-in schematics of the tip of the abdomen from 3 frames is shown at the bottom right. **g**, Mean oviDN ∆F/F aligned to the moment abdomen bending to lay an egg is complete. 43 traces from 9 cells in 8 flies (41 eggs). Light grey shading is ± s.e.m. for all panels in this figure. Behavior shown below. **h–n**, Mean oviDN ∆F/F and behavior aligned to events in the behavioral sequence shown in panel g. Locomotor speed is smoothed with a 5 s boxcar filter. **o**, Mean oviDN ∆F/F aligned to when abdomen bend is complete with all data points before the start of the search omitted from the average.

We developed an agarose-laden, rotatable, cylindrical treadmill on which a head-fixed fly could walk and lay eggs while we simultaneously performed either two-photon imaging or electrophysiological recording from neurons in the brain (Fig. 1d, Extended Data Fig. 2a–e and Methods). Each egg-laying wheel had regions with agarose interspersed with thin plastic barriers. The agarose substrates varied in their sucrose concentration (Fig. 1d, light and dark blue), but always contained 1.6% ethanol and 0.8% acetic acid, which simulate the chemical environment of a rotting fruit and thereby promote egg laying. We found that the egg-laying behavioural sequence measured on the wheel resembled that in free behaviour (Extended Data Fig. 2f,g). One difference was that flies on the wheel walked less vigorously during the search period (compare fly speed in Extended Data Figs. 1f and 2k), probably because they found it physically difficult to restart rotating the heavy wheel after standing still for a minute or more during ovulation (Methods). With head-fixed flies, we therefore often refer to the search period as the search/delay period.

**Extended Data Fig. 3:**
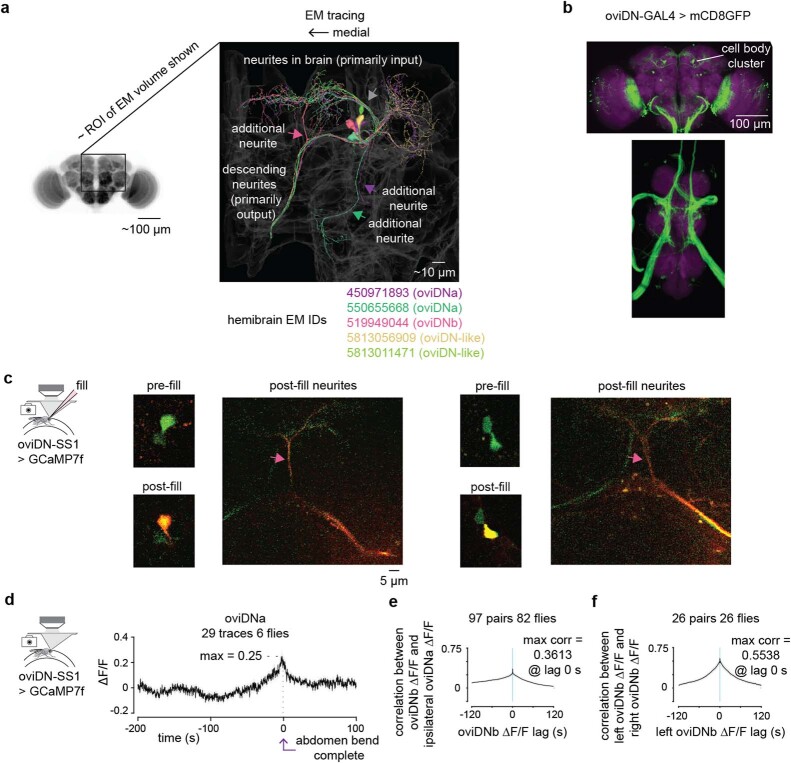
Anatomy and physiology of different oviDN types. **a**, Electron-microscopy (EM) skeletons^16^ and characterization of the 3 oviDN and 2 oviDN-like neurons per side. The branch labeled in grey is sometimes present in oviDNb^9^ and sometimes not (Fig. 1e). The 3 other arrows indicate neurites that are unique to either oviDNa or oviDNb. Visualization generated using Neuroglancer. Neuropil to left is only to schematize the approximate ROI shown in the EM. **b**, Average z-projection of oviDN-GAL4 in the brain (top) and ventral nerve cord (bottom). Green shows UAS-mCD8GFP expression in the targeted neurons and magenta represents a neuropil counterstain (Methods). **c**, Anatomy of oviDN-SS1 driving expression of GCaMP7f. The brighter of the two oviDN cell bodies was filled with Texas Red (Methods). The neurite labeled with a pink arrow in panel a was used to determine if the cell was oviDNb. All 6 of the brighter cells filled with Texas Red (from 6 separate flies) were oviDNb. Two examples are shown (representative individual z-slices). **d**, Mean oviDNa ∆F/F during individual egg-laying events. 29 traces from 7 cells in 6 flies (28 eggs). These data did not contribute to the traces in Fig. 1 (or any other figure), which were exclusively from oviDNb. Light grey shading is ± s.e.m. for all panels in this figure. **e**, Mean cross-correlation of ∆F/F between ipsilateral oviDNa and oviDNb cells imaged simultaneously. Traces from multiple, individual cell pairs are averaged. **f**, Mean cross-correlation of ∆F/F between contralateral oviDNb cells imaged simultaneously. Traces from multiple, individual cell pairs are averaged.

We decided to image the activity of oviposition descending neurons (oviDNs)^9^ during egg laying. These neurons appeared to be suitable candidates for informing the decision process because, when they are inhibited, egg laying is completely suppressed and when they are stimulated an egg is often laid^9^. Three oviDNs^9^ and two uncharacterized oviDN-like neurons are present on one side of the female fly brain, as anatomically characterized in the hemibrain connectome^16^ (totalling ten neurons per brain; Extended Data Fig. 3a). Each neuron primarily receives input in the brain and has synaptic outputs in the abdominal ganglion (Fig. 1e). We used two different driver lines to gain genetic access to oviDNs—oviDN-GAL4 and oviDN-SS1 (ref. ^9^). OviDN-GAL4 labels all oviDN and oviDN-like neurons (Extended Data Fig. 3b); OviDN-SS1 labels two of three oviDNs per side (cholinergic neurons named oviDNa and oviDNb)^9^ and neither of two oviDN-like neurons per side (Fig. 1f). In two-photon imaging experiments, unless otherwise stated, we used the oviDN-SS1 driver and targeted the oviDNb soma on one side of the brain; by targeting a single soma we could consistently image the same identified cell across all flies rather than intermixed neurites (Extended Data Fig. 3c).

## A rising signal in oviDNs

We imaged GCaMP7 (ref. ^17^) fluorescence from oviDNs during egg laying (Fig. 1g–l). We found that the oviDN ∆*F*/*F* signal dropped to its minimum value during ovulation and then peaked near the moment of the abdomen bend for egg deposition (Fig. 1g). In some cases we observed a monotonic rise (Fig. 1g, left and Supplementary Video 3) while in others the signal drifted up and down before reaching its peak (Fig. 1, right and Supplementary Video 4). The peak in the population-averaged ∆*F*/*F* signal was higher when we aligned the oviDN [Ca^2+^] signal with the moment when the abdomen finished bending to lay the egg (Fig. 1h,i) than when aligning with the moment that the egg became half-visible outside the fly (Extended Data Fig. 2l versus Extended Data Fig. 2m). On average, the [Ca^2+^] signal dipped when ovulation started (Fig. 1j) and reached a minimum when the abdomen was longest (Extended Data Fig. 2i). The average [Ca^2+^] signal then began to rise and returned to near baseline (∆*F*/*F* = 0 in our normalization; Methods) when ovulation was completed (that is, the beginning of the search/delay period; Fig. 1k). We often observed in individual traces an upward inflection in the [Ca^2+^] signal soon after the search/delay period began (Fig. 1g, right trace), which was evident as a small inflection in the mean trace (Fig. 1k, upward inflection just after time 0). The average [Ca^2+^] signal peaked at around 3 s before completion of abdomen bend for egg deposition (Fig. 1l)—that is, approximately when the bend was initiated. The average [Ca^2+^] signal returned to baseline after egg laying, while flies performed a second abdomen bend presumably to clean their ovipositor (Extended Data Fig. 2n).

**Extended Data Fig. 4:**
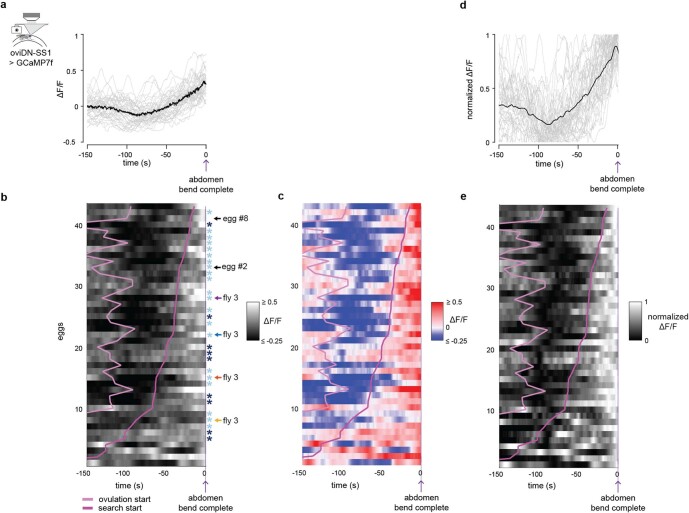
oviDN [Ca^2+^] traces from individual egg-laying events. **a**, OviDN ∆F/F showing all individual traces that were averaged in Fig. 1h (43 imaging traces from 41 egg-laying events associated with 9 cells in 8 flies). Grey lines are individual traces smoothed with a 5 s boxcar filter. Black line is the average of the non-smoothed individual traces. **b**, Each row represents a single egg-laying event. Rows have been ordered based on the search duration. Individual traces are smoothed as in panel a and behavioral annotations are overlayed. Individual traces corresponding to Fig. 1g and Fig. 1m (fly 3) are highlighted. Individual eggs that are part of the analysis in Fig. 4e, f are marked with an asterisk. **c**, Same as panel b but with a colormap where white is centered on a ∆F/F of 0 which is the average baseline in our normalization (Methods). Individual traces tend to (1) dip below baseline during ovulation (blue color after light pink/magenta line); (2) return to baseline around the time of search start (white color near dark pink/purple line); and (3) increase past baseline around or after the search start (red color after dark pink/purple line). These trends are captured by the average analysis presented in Fig. 1j–l. **d**, Normalized oviDN ∆F/F showing all individual traces in Fig. 1h (43 imaging traces from 41 egg-laying events associated with 9 cells in 8 flies). Grey lines are individual traces smoothed with a 5 s boxcar filter and then normalized such that the maximum and minimum ∆F/F in the 100 s window preceding the abdomen bend are set to 1 and 0, respectively. Black line is the average of the smoothed individual traces. **e**, Same as panel b but displaying ∆F/F normalized as in panel d.

**Extended Data Fig. 5:**
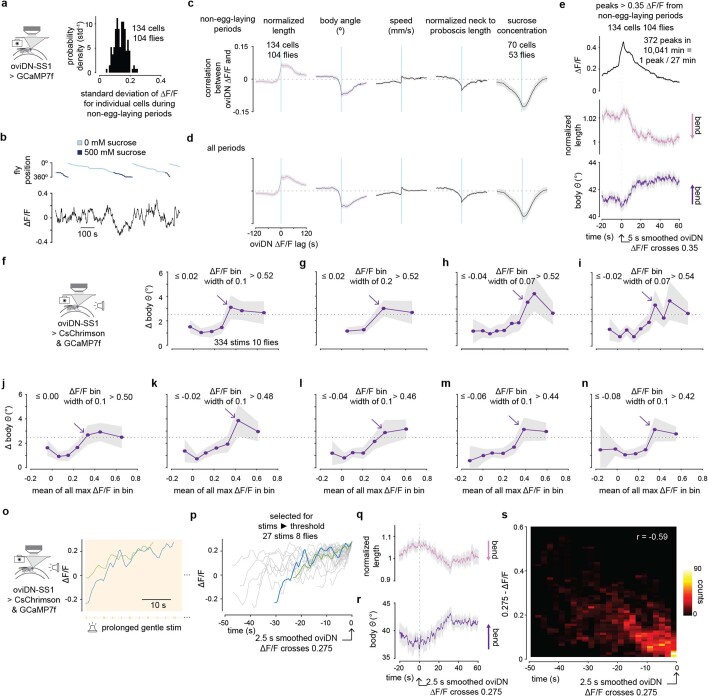
OviDN ∆F/F and fly behavior during non-egg-laying periods and during optogenetic stimulation. **a**, Standard deviation of oviDN ∆F/F for all data points > 5 min. away from egg deposition, i.e., ‘non-egg-laying periods’. **b**, Example trace of wheel position and oviDN ∆F/F during a non-egg-laying period (smoothed with a 2 s boxcar filter). This cell had a standard deviation in ∆F/F of 0.15. **c**, Mean cross-correlation of oviDN ∆F/F versus varied behavioral measures during non-egg-laying periods. Light grey shading is ± s.e.m. for all panels in this figure. For sucrose concentration correlations, only 0 vs. 500 mM sucrose wheels were analyzed (excluding 0 mM only wheels, for example), leaving 53/104 flies for analysis. **d**, Same as panel c, but including time periods near egg deposition (~372 additional minutes—i.e., ~4% additional sample points—are included compared to panel c). **e**, Mean oviDN ∆F/F and behavior during peaks in ∆F/F that occurred in non-egg-laying periods. We smoothed the ∆F/F signal with a 5 s boxcar filter and extracted peaks in the ∆F/F trace that exceeded 0.35 for > 1 s. We aligned these traces to the moment the ∆F/F signal crossed 0.35 in the 10 s before the peak. **f**, Change in mean body angle, replotted from Fig. 2h. Arrow indicates first bin with an abdomen angle change greater than 2.5° (indicated by dotted line). **g**, Same as panel f but with coarser binning. **h**, **i**, Same as panel f but with finer binning. **j-n**, Same as panel f but bins are shifted progressively by 0.02 leftward. In panels f to n, the first and last bin always include all the data points below and above that bin, respectively. The curve in panel l appears less step-like than the others; however, it is expected that as one progressively shifts the center point of the bins, one will find a position where the central bin straddles the putative threshold, yielding an intermediate y value for that bin. The fact that panels k and m appear more step like supports this explanation for panel l. **o**, Example traces of oviDN ∆F/F during prolonged, gentle CsChrimson stimulation (protocol described in Methods), smoothed with a 2.5 s boxcar filter. Traces are clipped once they reach a ∆F/F of 0.275. We used 0.275 as the threshold because it is slightly higher than the center of the 4^th^ bin in Fig. 2g, h (i.e., a conservative lower-bound estimate of the threshold). We use a conservative estimate for this analysis to capture as many relevant traces as possible. Note that for a variety of reasons, CsChrimson expressing flies may have a different threshold in terms of ∆F/F than flies not expressing CsChrimson (Methods). OviDN ∆F/F traces occasionally rise to threshold with this protocol. **p**, OviDN ∆F/F smoothed with a 2.5 s boxcar filter for all 27 stimulations (out of 127 total) that brought ∆F/F to threshold during the stimulation interval (the other 100 stimulations that did not bring ∆F/F to the threshold are not shown). The beginning of each trace is the beginning of stimulation. Colored lines are traces from panel o. A similar analysis in the inter-stimulation-interval (starting 10 s after the CsChrimson stimulation ended) only identifies 2 threshold crossing events indicating that the observed threshold crossing during stimulation was predominantly caused by the stimulation (data not shown). A similar analysis using data with the strongest 5 s stimulation intensity in Fig. 2f identifies 46 (out of 88 total) threshold crossing events indicating that is harder to achieve threshold crossing with the gentle prolonged stimulation despite the longer interval (data not shown). **q**, **r**, Change in mean body length and body angle for data shown in panel p, indicating that flies, on average, bend their abdomen proximal to the time of threshold crossing. **s**, Remaining ∆F/F until threshold is reached (y-axis) as a function of remaining time until threshold is reached (x-axis). The traces in panel p are sampled at 100 ms intervals to populate bin counts of the histogram. The negative correlation indicates that CsChrimson stimulation gradually brings the ∆F/F to threshold, rather than by inducing a spontaneous event, independent of the current ∆F/F, that brings ∆F/F to threshold.

The [Ca^2+^] rise was evident across multiple egg-laying events in single flies (Fig. 1m), reaching a qualitatively similar ∆*F*/*F* value of roughly 0.35 immediately before the abdomen bend for egg laying (Fig. 1n). In some flies we simultaneously imaged oviDNa and oviDNb, with both neuron types showing a similar rising signal (Extended Data Fig. 3d). When cross-correlating oviDNa and oviDNb GCaMP signals on the same side of the brain or oviDNb signals across both sides of the brain, we observed a peak with zero lag (Extended Data Fig. 3e,f). This observation supports a model in which all four oviDNs in the oviDN-SS1 line exhibit the same first-order calcium dynamics during egg laying. Thus, in our recordings of single oviDNs, when we observe an occasional ∆*F*/*F* peak with no egg or an egg without a peak in the ∆*F*/*F* signal (Fig. 1m and Extended Data Fig. 4), this may be because the functionally relevant signal is a population-level one across all six oviDNs. Aspects of this ∆*F*/*F* variability might also reflect technical considerations associated with stable acquisition of long [Ca^2+^] measurements from a single, tiny, soma in a behaving fly. During non-egg-laying periods, the oviDN ∆*F*/*F* signal still correlated with abdominal movements and locomotion (Extended Data Fig. 5a–d). Approximately once every 30 min the oviDN ∆*F*/*F* signal reached around 0.35 without ovulation having occurred beforehand, and at these moments the fly exhibited an abdomen bend that yielded no egg (Extended Data Fig. 5e). In sum, oviDNs express a signal whose dynamics correlate with the behavioural sequence of *Drosophila* egg laying, drifting up and down during the search period until a consistent level is reached just before egg deposition. These dynamics suggested that a rise-to-threshold process governs *Drosophila* egg-laying behaviour, a hypothesis that we next tested with optogenetics.

## Optogenetics supports a threshold

**Fig. 2:**
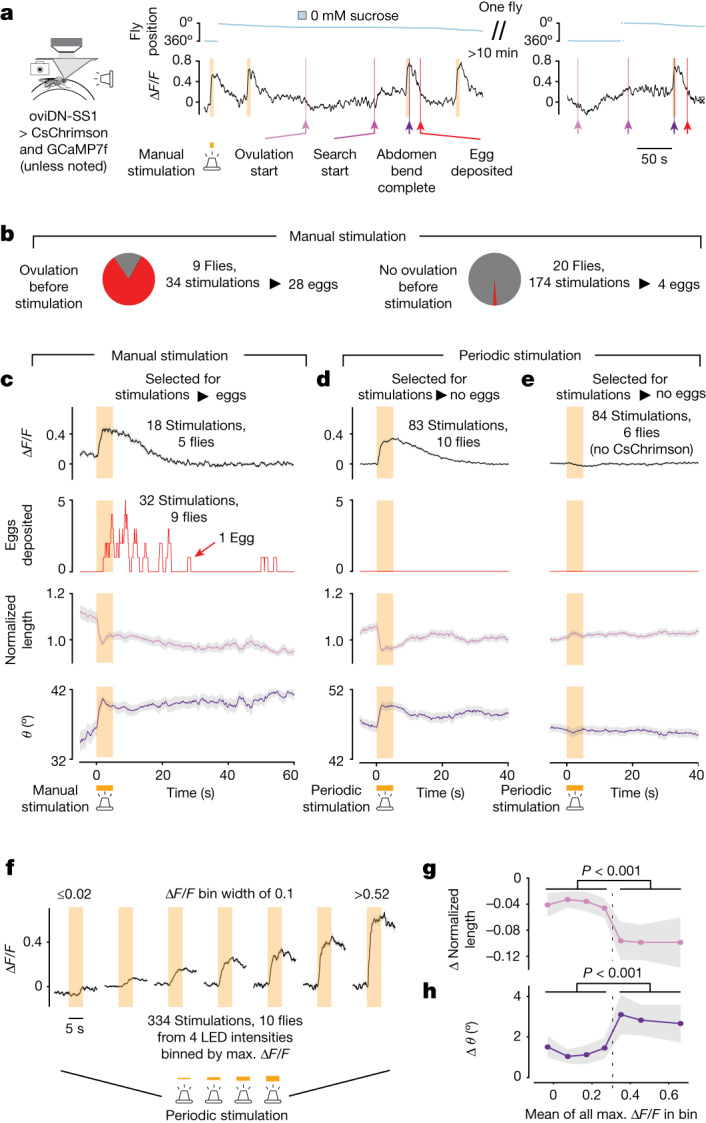
Evidence for a threshold in the ability of oviDNs to trigger the egg-deposition motor programme. **a**, oviDN ∆*F*/*F* and behaviour during high-intensity 5 s CsChrimson stimulation. ∆*F*/*F* is smoothed with a 2 s boxcar filter. **b**, High-intensity stimulations separated on the basis of whether ovulation was observed previously. Stimulations resulting in eggs were defined as those in which egg deposition occurred within 60 s of light onset. All four eggs without ovulation observed previously were from the first stimulation of a fly, and ovulation may have occurred before the session. **c**, Mean oviDN ∆*F*/*F* and behaviour for manually triggered, high-intensity stimulations that resulted in eggs. Light grey shading represents ±s.e.m. throughout. Behaviour, 32 stimulations in nine flies; ∆*F*/*F*, 18 stimulations in five flies. Differences in number of traces are explained in Methods. The peak in oviDN ∆F/F slightly lags behind initiation of the abdomen bend, potentially because [Ca^2+^] at the synaptic active zones rises faster than at the soma with optogenetic stimulation. **d**, Mean oviDN ∆*F*/*F* and behaviour for periodically triggered, high-intensity stimulations that did not result in eggs. Five of 88 stimulations that resulted in eggs are not shown so that changes independent of egg deposition could be analysed. **e**, Same as **d** but with flies not expressing CsChrimson (0 of 84 stimulations → eggs). **f**, oviDN ∆*F*/*F* during stimulation binned by maximum ∆*F*/*F* 1–3 s after start of stimulation. Four light intensities were triggered periodically. Stimulations were included regardless of whether egg deposition occurred (nine of 334 stimulations → eggs). The first and last bins include data below 0.02 and above 0.52, respectively. **g**,**h**, Change in mean body length (**g**) and body angle (**h**) for each of the bins in **f**. Mean behavioural signal 2–4 s after start of stimulation was subtracted from mean behavioural signal 0–2 s before stimulation. Two-sided Wilcoxon rank-sum test, *P* = 7.2 × 10^–4^ and 5.0 × 10^–4^. LED, light-emitting diode.

To test whether a neural activity threshold triggers the egg-deposition motor programme, we coexpressed in oviDNs GCaMP7f and the light-gated ion channel CsChrimson^18^. We measured oviDN ∆*F*/*F* and fly behaviour while providing 5-s-long, high-intensity light pulses (Methods). Stimulations after ovulation typically yielded an abdomen bend and egg deposition (Fig. 2a,b and Supplementary Video 5). When we averaged [Ca^2+^] and behavioural signals around the time of stimulations that yielded an egg we observed an increase in ∆*F*/*F* in the oviDN, a synchronous abdomen bend and—with more variable latency—egg deposition (Fig. 2c).

In our initial experiments we stimulated oviDNs at user-defined moments, sometimes purposefully waiting for flies to finish ovulating before stimulating (Methods). In later experiments we performed regularly spaced stimulations in flies expressing or not expressing CsChrimson, independent of the flies’ ovulation status. Flies expressing CsChrimson bent their abdomen, on average, even on stimulation pulses that did not result in egg deposition (Fig. 2d), whereas control flies did not bend their abdomen (Fig. 2e). We interpret this result—alongside the observation that flies tended to bend their abdomen when oviDN ∆*F*/*F* was spontaneously high without previous ovulation (Extended Data Fig. 5e)—to mean that they initiate the egg-deposition motor programme when a neural process reflected in the oviDN [Ca^2+^] signal reaches a certain level. If an egg is available in the uterus, egg deposition occurs—although with temporal variability that may be related to sensory feedback signals in the uterus^12^ or motor aspects of how eggs are released^13^. The temporal variability in egg deposition was qualitatively similar in optogenetically stimulated (Fig. 2c) and spontaneous (Fig. 1h) egg laying in head-fixed flies.

To quantitatively assess whether the egg-deposition motor programme is initiated in an all-or-nothing fashion when neural activity crosses a threshold, we stimulated oviDNs at a regular interval while cycling through four different intensities of light. We assigned each stimulation trial to one of seven bins depending on the oviDN ∆*F*/*F* maximum on that stimulation pulse (Fig. 2f). We found that, when our stimulation pulse induced ∆*F*/*F* changes of approximately 0.32 or higher, the pulse produced large mean abdomen bends and, when our stimulation pulse induced ∆*F*/*F* changes below that level, the pulse did not induce such bends (Fig. 2g,h). This bimodality was robust to how we binned ∆*F*/*F* responses (Extended Data Fig. 5f–n). (Note that although the ∆*F*/*F* threshold value here is similar, but not identical, to that observed during spontaneous egg laying, any such quantitative comparison is not necessarily biologically meaningful (Methods).) We also found supportive evidence for a threshold when we provided gentle stimulation to oviDNs for tens of seconds and correlated the moment at which oviDN ∆*F*/*F* reached a common value with when an abdomen bend was observed (Extended Data Fig. 5o–s). Altogether, these data support the hypothesis that a threshold level of activity initiates the egg-deposition motor programme in an all-or-nothing fashion.

**Extended Data Fig. 6:**
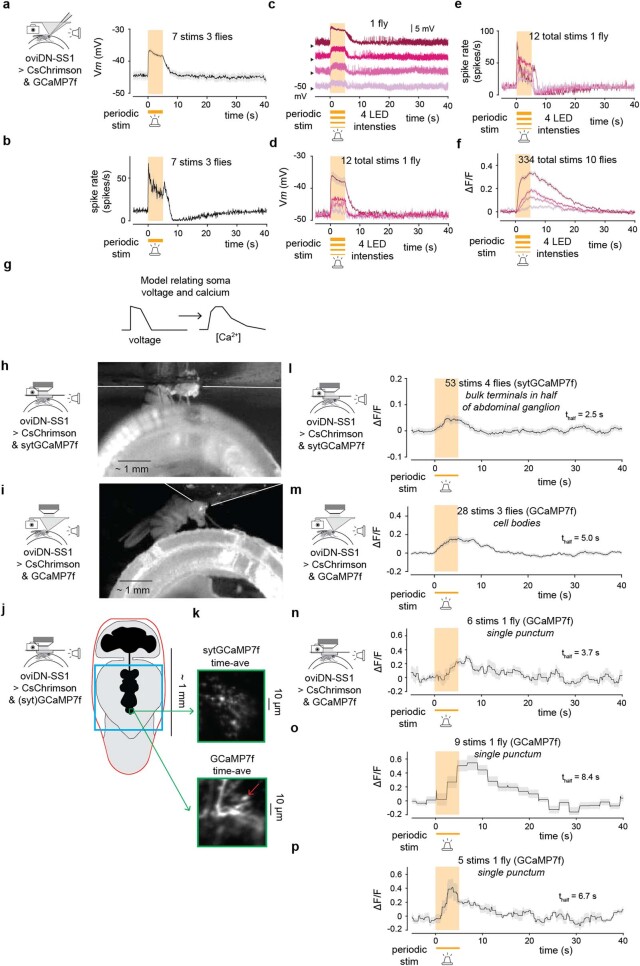
[Ca^2+^] changes in the oviDN soma and presynaptic terminals lag changes in electrical activity. **a**, Mean oviDN V*m* during periodically triggered high-intensity 5 s CsChrimson stimulations. Light grey shading is ± s.e.m. for all panels in this figure. **b**, Mean oviDN spike rate during periodically triggered high-intensity 5 s CsChrimson stimulations. **c**, OviDN single-trial V*m* traces during periodically triggered 5 s CsChrimson stimulations at four different intensities in the same fly. Intensities are the same as in Fig. 2f–h. Traces have been shifted on the y-axis for clarity, with –50 mV indicated for each trace (black arrowhead). **d**, Mean oviDN V*m* during periodically triggered 5 s CsChrimson stimulations at four different intensities (same fly as panel c). **e**, Mean oviDN spike rate during periodically triggered 5 s CsChrimson stimulations at four different intensities (same fly as panel c). **f**, Mean oviDN ∆F/F during periodically triggered 5 s CsChrimson stimulations at four different intensities (same data as Fig. 2f). **g**, Graphical model of the link between voltage and calcium in oviDN somas using evidence from panels a to f. Increases in voltage lead to slower increases in calcium and decreases in voltage lead to slower decreases in calcium. To first order calcium ∆F/F signals appear to be a low-pass filtered, delayed version of the voltage changes observed. Since CsChrimson does not permeate calcium^79^, changes in [Ca^2+^] observed during stimulation are likely due to opening of voltage-gated calcium channels. **h**, Preparation to image oviDN presynaptic terminals in the abdominal ganglion of the ventral nerve cord (Methods). **i**, Standard preparation for imaging the oviDN cell body in brain. **j**, Schematic (top view) of the holder in panel h. An outline of the hole in which the thorax, head, and anterior abdomen are inserted is shown in red. The dissected region is indicated in blue. A typical calcium imaging region is shown in green. **k**, Z-projections of representative calcium imaging regions. Compare to region indicated by green arrow in Fig. 1e. sytGCaMP7f^80,81^ was used to bias GCaMP expression to presynaptic compartments for *bulk imaging* of presynaptic terminals. Note that sytGCaMP biases GCaMP expression to terminals, but not necessarily to active zones^81^. Red arrow points to the punctum quantified in panel n. **l**, Mean oviDN ∆F/F in bulk presynaptic compartments during periodically triggered CsChrimson stimulation, using 2^nd^ lowest intensity from panels c to f. A low stimulation intensity was applied such that subthreshold calcium accumulation could be investigated. Presynaptic compartments from oviDNa and oviDNb could not be distinguished and are thus averaged together. **m**, Mean oviDN ∆F/F in cell bodies during periodically triggered CsChrimson stimulation. To aide comparison with panel l, this experiment was done at a similar time, with similar conditions (Methods), and with ROIs encompassing both oviDNa and oviDNb cell bodies. **n–p**, Mean oviDN ∆F/F in selected single presynaptic compartments, from three different flies, during periodically triggered CsChrimson stimulation using the subthreshold intensity in panels l and m. ROIs were drawn around individual puncta in GCaMP7f expressing flies, which had a stronger florescence signal than sytGCaMP7f flies.

In these experiments we measured [Ca^2+^] in the oviDN soma. Somatic [Ca^2+^] is often thought of as a proxy for a cell’s spike rate^19^. To gain insight into the relationship between membrane potential (*V*_m_), spike rate and [Ca^2+^] in oviDNs, we activated CsChrimson while performing either whole-cell patch-clamp recordings or calcium imaging at the oviDN soma (Extended Data Fig. 6a–g). The oviDN spike rate and *V*_m_ rose and fell quickly with stimulation (around 400 ms half-decay time for both) whereas somatic [Ca^2+^] changed much more slowly (roughly 5.7 s half-decay time in the ∆*F*/*F* signal; Extended Data Fig. 6d–f and Methods). Given these slow [Ca^2+^] dynamics, the ∆*F*/*F* threshold that we measured at the soma may not represent a consistent spike-rate threshold in the same cell, which raises the question of how the somatic signal we analysed induces behaviour. One possibility is that the [Ca^2+^] signal in the oviDN soma acts as a proxy for a functionally relevant rise-to-threshold process in the abdominal ganglion, perhaps in the oviDN axon terminals. Consistent with this possibility, when we imaged GCaMP fluorescence in the axonal terminals of oviDNs during CsChrimson stimulation we also observed relatively slow [Ca^2+^] dynamics (Extended Data Fig. 6h–p and Supplementary Discussion). Thus, the rising [Ca^2+^] signal in the soma might reflect a similarly rising [Ca^2+^] signal in the axon terminals, with a biochemical process in the presynaptic terminals of oviDNs potentially reading out the rising [Ca^2+^] signal with a sharp nonlinearity to trigger the egg-laying motor programme. Alternatively, oviDNs may transmit a graded synaptic signal to their postsynaptic partners, with the threshold implemented downstream of oviDNs. Additional work will be needed to test these hypotheses.

## Searching for a substrate of high value

**Fig. 3:**
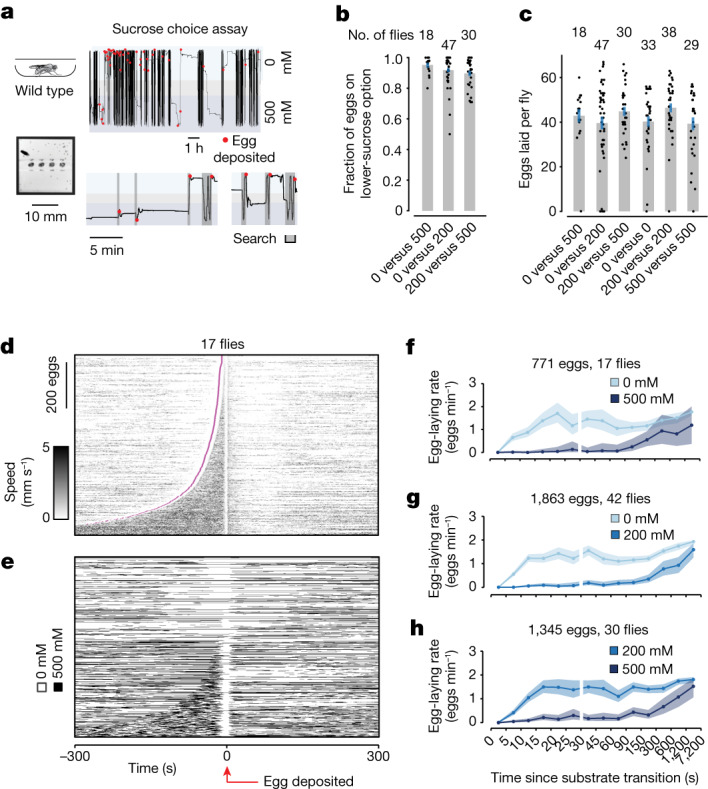
Flies search for an egg-deposition site with high relative value in the time period when the oviDN [Ca^2+^] signal rises. **a**, *Y* position and egg-deposition events from a fly in a high-throughput egg-laying choice chamber^20^. **b**, Fraction of eggs on the lower-sucrose option with 95% confidence interval. *X* axis indicates sucrose concentration (mM). One dot represents one fly. **c**, Eggs laid per fly. Mean ±s.e.m. indicated. One dot represents one fly. **d**, Each row represents a single egg-laying event in a 0 versus 500 mM sucrose chamber, aligned to egg deposition, with the fly’s speed indicated by colour intensity. Rows have been ordered based on the search duration; start of the search period is in magenta. Eighteen flies were tested, one of which did not lay eggs. **e**, Same data as in **d**, but the substrate on which the fly was residing is indicated by white and black pixels. **f**–**h**, Mean egg-laying rate during the search period aligned to a transition from higher to lower sucrose (lighter blues) or lower to higher sucrose (darker blues) in three separate choice conditions (0 versus 500 mM (**f**), 0 versus 200 mM (**g**) and 200 versus 500 mM (**h**)), with 90% confidence intervals (Methods): 771 eggs from 17 flies (**f**, 18 flies tested of which one did not lay eggs), 1,863 eggs from 42 flies (**g**, 47 flies tested of which five did not lay eggs) and 1,345 eggs from 30 flies (**h**, 30 flies tested). Egg-laying rate requires around 10 s to reach maximum after a fly transitions to the higher-relative-value option, at least partially because flies do not lay eggs on the (approximately) 2.5 mm plastic boundary between substrates (Extended Data Fig. 7e,f) and because there is a delay of about 3 s between when the fly bends its abdomen and deposits the egg (Extended Data Fig. 7g and Fig. 1c). Thus, the fly’s internal sense of relative value probably changes more rapidly after a transition than the slowly increasing egg-laying-rate curve would suggest.

If a threshold triggers initiation of the egg-deposition motor programme, might substrate quality modulate oviDN activity to influence when threshold is reached and thus where an egg is laid? We analysed the behaviour of freely walking flies to better understand how they use substrate experiences during their search—that is, the time period after ovulation and before egg deposition—to guide egg-laying decisions. Specifically, we quantified where flies laid eggs within custom, high-throughput behavioural chambers with two different substrate options^20^ (Fig. 3a, Extended Data Fig. 1b, Supplementary Video 6 and Methods).

We observed, in line with past work^7,8^, that *Drosophila melanogaster* target the majority of their eggs to substrates with lower, not higher, concentrations of sucrose (Fig. 3b). This bias makes sense in light of the fact that *D. melanogaster* prefer to lay eggs on rotting or fermenting fruit^21^, and a soft substrate with clearly detectable ethanol and relatively low levels of sucrose^22^ mimics the portion of a rotting fruit where fermentation (conversion of sugar to alcohol) is actively taking place. Beyond simply preferring low sucrose, we further replicated past findings arguing that sucrose-based choice is a relative-value decision^7,8^. That is, flies strongly bias egg laying to the lower of two sucrose options rather than preferring an absolute sucrose concentration. For example, they laid over 90% of eggs on the 0 mM option in 0 versus 200 mM chambers and over 90% of eggs on the 200 mM option—the previously avoided substrate—in 200 versus 500 mM chambers (Fig. 3b). Flies laid a similar total number of eggs in all chambers^7,8^ (Fig. 3c).

**Extended Data Fig. 7:**
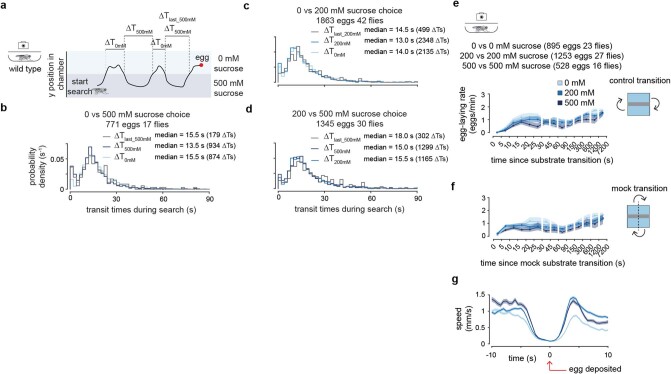
Evidence against flies using spatial information in substrate search and against a feeding-on-higher-sucrose related explanation for substrate preferences in our free behavior chambers, alongside controls for the egg-laying rate function. **a**, Schematic of a fly searching for an egg deposition site in a 0 vs. 500 mM chamber. ∆T_0mM_ and ∆T_500mM_ are all the intervals of time that a fly spent on 0 or 500 mM, respectively, during an egg-laying search period. ∆T_last_500mM_ is the last transit interval through 500 mM for eggs deposited on 0 mM. If a fly were positionally avoiding sucrose, ∆T_500mM_ would be less than ∆T_0mM_. If a fly were to use spatial information during the search period—by taking a shortcut to get to the preferred 0 mM substrate at the end of a search—∆T_last_500mM_ would be less than ∆T_0mM_ and ∆T_500mM_. If a fly were feeding on the higher sucrose substrate—and pausing as flies do when they feed^82^—∆T_500mM_ would be larger than ∆T_0mM_. **b-d**, ∆T_lower_sucrose_, ∆T_higher_sucrose_, and ∆T_last_higher_sucrose_ distributions for three different sucrose choice chambers. ∆T_higher_sucrose_ is not less than ∆T_lower_sucrose_ suggesting that flies are not positionally avoiding the higher sucrose option. ∆T_last_higher_sucrose_ is not detectably smaller than ∆T_0mM_ or ∆T_500mM_ suggesting that flies are not taking a shortcut—and thus not manifesting use of spatial information—at the end of the search. It is possible that flies use spatial information to guide the search in conditions with visible landmarks or where they perform less thigmotaxis (edge-hugging); our flies largely edge-hugged as they traversed the chamber. All experiments in this study were conducted in darkness. Note that our time-domain model for egg laying (Fig. 4a) could be readily augmented with spatial knowledge in that flies could putatively use their spatial sense to control which substrate they visit which would then impact their egg-laying drive. ∆T_higher_sucrose_ is not larger than ∆T_lower_sucrose_ indicating that flies are not pausing only on the higher sucrose substrate. We interpret this result to mean that flies are not suppressing egg deposition because of extensive feeding on the sucrose substrates. In addition, we did not notice additional proboscis extension on higher sucrose when we spent hours inspecting each video to annotate the egg deposition times. Note that our flies were very well fed before entering the chamber, which could have minimized this effect (Methods). 771 eggs from 17 flies (18 flies tested and 1 did not lay eggs), 1863 eggs from 42 flies (47 flies tested and 5 did not lay eggs), and 1345 eggs from 30 flies (30 flies tested), respectively. **e**, Mean egg-laying rates during the search period after a fly transitions across the plastic barrier in a single-option chamber, meaning that there is either 0 mM sucrose on both sides, 200 mM sucrose on both sides, or 500 mM sucrose on both sides. 90% confidence interval shaded. Egg-laying rates on the three different sucrose concentrations are similar in single-option chambers. The slightly higher egg-laying rates on lower sucrose is consistent with a possible, slight, innate preference for lower sucrose, which interacts with a much more prominent relative-value assessment of sucrose that governs egg laying rates (Fig. 3f–h). 895 eggs from 23 flies (24 flies tested and 1 laid no eggs), 1253 eggs from 27 flies (27 flies tested), and 528 eggs from 16 flies (17 flies tested and 1 laid no eggs) for 0 vs. 0, 200 vs. 200, and 500 vs. 500 mM chambers, respectively. **f**, Mean egg-laying rate during the search after a fly transitions across a mock vertical line. 90% confidence interval shaded. Same data as in panel e. The 5–10 s bin in this analysis has a higher egg laying rate than in the analysis from panel e, suggesting that part of the delay in egg laying after a transition is due to flies not laying eggs on the plastic barrier. **g**, Mean locomotor speed with ± s.e.m. shaded. A ~3 s delay exists between when a fly pauses and bends its abdomen to lay an egg till when an egg is deposited. This ~3 s latency is at least part of the reason why even the data in panel f do not show high egg laying rates in the 0–5 s bin. Analyzing the same data as in panels e-f.

In these high-throughput chambers we did not have the spatial resolution to clearly detect abdominal elongations and scrunches (Extended Data Fig. 1b,c and Methods). However, we could still detect ovulation and thus when flies start to search immediately thereafter, because they stand still for about 1 min when they ovulate (Extended Data Fig. 1d–f and Methods). We could also denote the end of the search period as the moment when an egg was half-way out of the ovipositor, which consistently follows the final abdomen bend for egg laying by only a few seconds in these chambers (Methods). The duration of the search period was highly variable (Fig. 3d). Flies laid more eggs on the lower-sucrose option despite spending appreciable time on the higher option during the search epoch^8^ (Fig. 3e). Specifically, in 0 versus 500 mM chambers, 95% (734 of 771) of eggs were laid on 0 mM whereas only 77% (592 of 771) of search periods started on 0 mM (*P* < 0.001; Methods). (More search periods started on 0 mM than 500 mM because ovulation tended to occur soon after the previous egg-laying event (Extended Data Fig. 1d) and egg laying tended to occur on 0 mM.) We additionally noticed that, when flies started the search on 500 mM, they frequently left this substrate while searching (83%, 149 of 179) but when they started their search on 0 mM they left less often (36%, 212 of 592; *P* < 0.001; Methods). Leaving a higher-sucrose substrate more often at the onset of search is not an intrinsic property of the substrate, because flies left substrate islands at a similar rate in 500 versus 500 and 0 versus 0 mM chambers (299 of 528, 57% and 441 of 895, 49%, respectively). Because sucrose cannot be sensed at a distance, we conclude that flies retain information about the substrate options available to them from experiences outside of the current search period and use this information to regulate the current search. We tested for the possibility of flies using spatial memories to guide their egg-laying behaviour in our chambers but we could not find supportive evidence (Extended Data Fig. 7a–d). We also did not find evidence that flies were pausing to feed on the higher-sucrose substrate while searching, suggesting that in our experiments a competing feeding drive is not the reason for suppression of egg laying on higher-sucrose substrates (Extended Data Fig. 7a–d).

We noticed that flies would occasionally lay eggs on the higher-sucrose option if a few minutes had elapsed since they last visited the preferred, lower-sucrose option (Fig. 3a bottom, first two eggs). To quantify this observation we calculated the egg-laying rate during the search period as a function of time since the last substrate transition (regardless of whether the last transition occurred in the current search period or previously; Methods). Flies in 0 versus 500 mM sucrose choice chambers strongly inhibited egg laying on 500 mM if they had visited the 0 mM option within the previous 2 min or so (Fig. 3f). After about 2 min, however, the egg-laying rate on 500 mM began to increase gradually, approaching—albeit not completely—that on 0 mM at the 2 h time point. One interpretation of this egg-laying-rate plot is that the relative value of the 500 mM substrate gradually increased over time, eventually approaching the value of the 0 mM substrate (if 0 mM is not revisited). This phenomenon was also evident in 0 versus 200 mM and 200 versus 500 mM chambers (Fig. 3g,h).

## Substrate value alters oviDN physiology

**Fig. 4:**
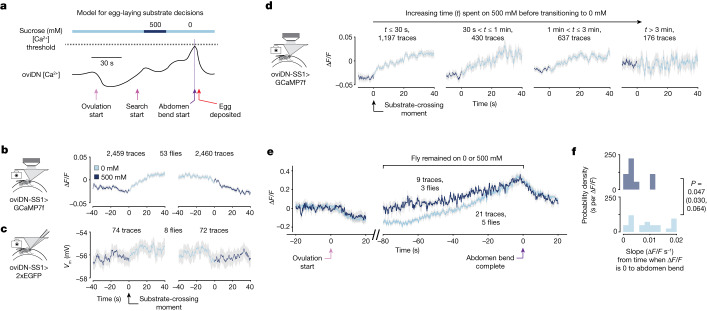
Relative value of the current egg-laying option influences the subthreshold physiology of oviDNs to impact when threshold is reached. **a**, Schematic model relating oviDN signal to substrate decisions. **b**, Mean oviDN ∆*F*/*F* during substrate transitions. Light grey shading denotes ±s.e.m. throughout. In total, 2,459 and 2,460 traces from 70 cells in 53 flies (1,911 and 1,922 transitions); 1,911 transitions yielded 2,459 traces because we sometimes imaged oviDNb on both sides of the brain. **c**, Mean oviDN *V*_m_ during transitions; 74 and 72 traces from eight cells in eight flies (74 and 72 transitions). Traces were smoothed using a 666 ms boxcar filter to aid comparison to ∆*F*/*F*, which was acquired at around 1.5 Hz. **d**, Mean oviDN ∆*F*/*F* during transitions split based on the amount of time the fly spent on 500 mM before entering 0 mM; 1,197, 430, 637 and 176 traces from 70 cells in 53 flies (914, 347, 486 and 148 transitions, respectively). **e**, Mean oviDN ∆*F*/*F* for egg-laying events where the fly remained on 0 or 500 mM for the 80 s window before and including egg deposition. An increased ∆*F*/*F* baseline of roughly 0.02 exists for 0 mM before ovulation; 0 mM, 21 traces from five cells in five flies (21 eggs); 500 mM, nine traces from four cells in three flies (seven eggs). **f**, Probability densities of individual oviDN ∆F/F slopes from traces averaged in **e**. Individual ∆*F*/*F* values were smoothed with a 5 s boxcar filter before calculating the net slope from when ∆*F*/*F* first reached 0 after the signal minimum (which occurs during ovulation) to 3.3 s before abdomen bend was complete—which is when, on average, abdomen bend starts (Fig. 1l). *P* values were calculated using the two-sided Wilcoxon rank-sum test. For additional information on these calculations see Methods.

How might the rise-to-threshold process evident in oviDN [Ca^2+^] guide flies to lay most of their eggs on substrates with high relative value? We hypothesized that, when flies are on a high-value substrate, the oviDN [Ca^2+^] signal might rise briskly and, when they are on a low-value substrate, it might rise more slowly or even fall, thus creating time for the fly to find a better option before threshold is reached (Fig. 4a).

**Extended Data Fig. 8:**
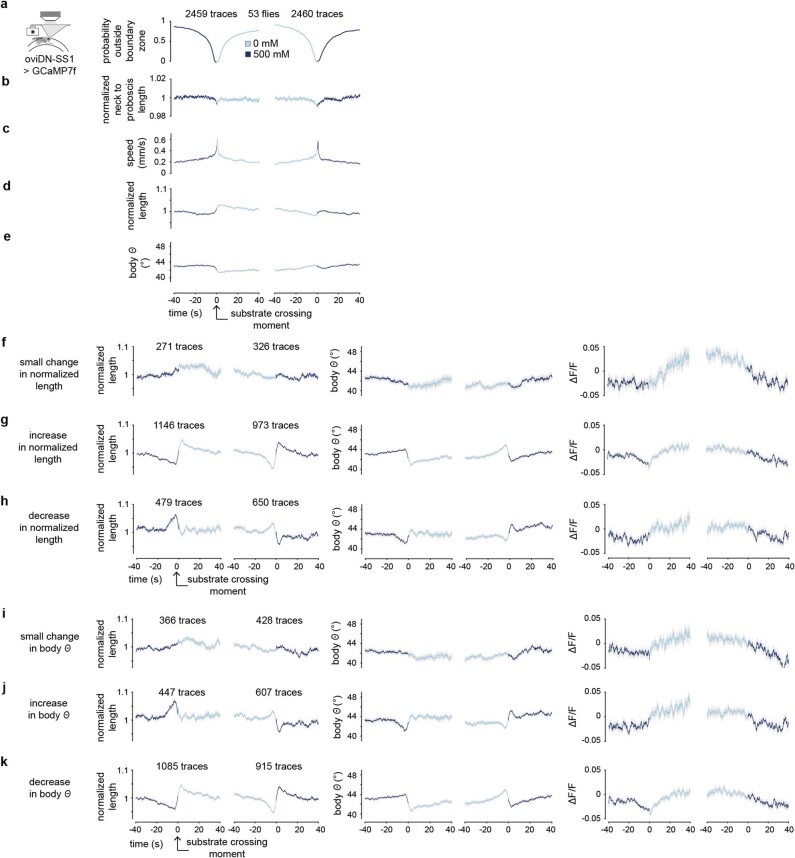
Changes in oviDN ∆F/F during substrate transitions are not due to consistent, detectable, changes in behavior. **a**, We detected substrate crossing moments on the egg-laying wheel, and aligned behavioral data to these moments: 2459 and 2460 traces from 70 cells in 53 flies (1911 and 1922 transitions). Plotted here is the probability that a fly’s centroid is located > 2 mm away from the boundary between two substrates (y axis), as a function of time from the substrate crossing (x axis). For a 2.5 mm fly, not being in the 2 mm region surrounding the boundary corresponds to the front or back of the fly being 0.75 mm away from the midpoint of the 1 mm plastic barrier between substrates. These traces highlight that it takes flies ~10–20 s, on average, to completely cross the midline which is important to keep in mind when interpreting neural signals aligned to substrate crossing events. **b**, Mean neck to proboscis length during substrate transitions. Light grey shading is ± s.e.m. for all panels in this figure. **c**, Mean locomotor speed during substrate transitions. **d**, Mean body length during substrate transitions. **e**, Mean body angle during substrate transitions. **f**, Mean body length, body angle, and oviDN ∆F/F during the subset of substrate transitions where there was a small change in body length. The mean body length in the 4 s after and before a substrate transition were subtracted. If the absolute value of this difference was less than 0.01, then the change was considered small. **g**, Same as panel f, except selecting for substrate transitions where the difference was greater than 0.01. **h**, Same as panel f, except selecting for substrate transitions where the difference was less than −0.01. The sum of the number of traces in panels f-h is less than panel a because during some substrate transitions the body length and/or angle was not possible to accurately calculate using DeepLabCut (Methods). **i–k**, Same as panels f-h, except comparing body angle and using a threshold of 0.5°. Proboscis length and fly speed (panels b-c) do not consistently change during substrate transitions and therefore do not explain the changes in oviDN ∆F/F. Body length and body angle do change, on average, during substrate transitions (panels d-e). However, these changes cannot fully explain the changes in oviDN ∆F/F (panels f-k). That is, regardless of the change in body length or body angle, the oviDN ∆F/F consistently changes with sucrose concentration (albeit with some modulations related to body length and angle).

**Extended Data Fig. 9:**
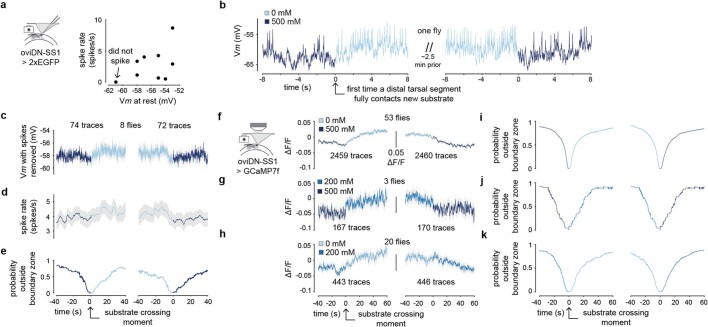
OviDN electrical activity during substrate transitions and additional evidence for oviDN ∆F/F tracking relative value during substrate transitions. **a**, oviDN spike rate versus V*m* at rest. **b**, V*m* during two substrate transitions from the same fly. These sample traces have more pronounced V*m* changes than is typical. **c**, Mean oviDN V*m* after removal of spikes during substrate transitions (Methods). 74 and 72 traces from 8 cells in 8 flies (74 and 72 transitions). Light grey shading is ± s.e.m. for all panels in this figure. **d**, Mean oviDN spike rate during substrate transitions. **e**, Same as Extended Data Fig. 8a but for the behavior of the fly during this electrophysiology dataset. **f**, Mean oviDN ∆F/F during substrate transitions from 500 to 0 mM and 0 to 500 mM. 2459 and 2460 traces from 70 cells in 53 flies (1911 and 1922 transitions). **g**, Mean oviDN ∆F/F during substrate transitions from 500 to 200 mM and 200 to 500 mM. 167 and 170 traces from 5 cells in 3 flies (105 and 109 transitions). **h**, Mean oviDN ∆F/F during substrate transitions from 200 to 0 mM and 0 to 200 mM. 443 and 446 traces from 20 cells in 20 flies (443 and 446 transitions). In panels f-h, note that all changes are on the order of 0.05 ∆F/F regardless of the absolute sucrose concentration, consistent with a relative value calculation. **i–k**, Same as Extended Data Fig. 8a but for the behavior of the fly during the datasets in panels f-h, shown to the left.

To test this idea we analysed how the oviDN ∆*F*/*F* signal changed as flies transitioned across substrates on the egg-laying wheel. On wheels with 0 and 500 mM sucrose options we observed a mean increase in ∆*F*/*F* after flies walked onto the higher-relative-value substrate (500 → 0 mM transitions) and a mean decrease after they transitioned to the lower-relative-value substrate (0 → 500 mM transitions) (Fig. 4b). This result was not explained by differences in feeding, locomotor speed or abdomen movements across the two options (Extended Data Fig. 8). We observed similar, but qualitatively faster, changes in oviDN activity with substrate transitions at the level of *V*_m_ (Fig. 4c) and spike rate (Extended Data Fig. 9a–e).

If oviDN [Ca^2+^] tracks the relative value of substrates, rather than just sucrose concentration, one might expect that oviDN ∆*F*/*F* would gradually increase on the 500 mM option because that option becomes more acceptable over several minutes. Indeed, when we split 500 to 0 mM substrate transitions into four groups—depending on the time spent on 500 mM before the transition—we found that the mean, ‘baseline’, ∆*F*/*F* on 500 mM became progressively higher. After more than 3 min on 500 mM, the mean ∆*F*/*F* on 500 and 0 mM became indistinguishable (Fig. 4d). It is intriguing that this slow increase in oviDN mean [Ca^2+^] in flies residing on a 500 mM substrate occurred on a time scale of minutes, which roughly matches the time scale over which egg-laying rates recover in flies residing on 500 mM in free behaviour (compare Fig. 4d with Fig. 3f). Consistent with the notion that the mean oviDN [Ca^2+^] signal tracks relative value and not just sucrose concentration, the magnitude of the average ∆*F*/*F* changes during substrate transitions in 0 versus 500 mM wheels, 0 versus 200 mM wheels and 200 versus 500 mM wheels were similar (Extended Data Fig. 9f–k).

We hypothesized that excitatory inputs associated with the relative value of the current substrate interact with additional excitatory drive associated with the search state. These two inputs ultimately drive oviDN activity to hit threshold, inducing egg laying. One prediction of this model is that the oviDN [Ca^2+^] signal should have a lower propensity to rise on the less valued substrate because of reduced drive from putative relative-value inputs, and a higher propensity to rise on more valued substrates. Although the number of eggs available for analysis was very low, we found that the mean slope of oviDN ∆*F*/*F* rise toward threshold was shallower on the lower-relative-value substrate than on the higher one (Fig. 4e). A change in slope was also evident, to near statistical significance, in an analysis of individual traces (Fig. 4f). The path to threshold of individual traces was not as gradual as in the average trace, often containing acute upward and downward fluctuations (Fig. 1g,m and Extended Data Fig. 4). These fluctuations could reflect internal gating of when substrate value inputs impact oviDN physiology, or other factors that influence egg laying. Indeed, such fluctuations may underlie the sizeable variability in search duration we observed in freely behaving flies regardless of whether they were presented with one or more substrate options (Figs. 1c and 3d). Note that, in free behaviour, we would expect modulations of the oviDN signal to show even more marked upward or downward adjustments than those in Fig. 4e because, unlike head-fixed flies, freely walking flies will transition more often between low- and high-relative-value substrates during search.

## Hyperpolarization of oviDNs alters choice

Given the above framework for how the oviDN signal relates to egg-laying substrate choice (Fig. 4a), we asked whether we might be able to perturb oviDNs in a manner that would cause flies to lay even more eggs than normal on the option with higher relative value. Specifically, we reasoned that gentle hyperpolarization of all oviDNs (using the oviDN-GAL4 line) could lengthen the time required for the decision process to reach threshold, providing flies with more time than usual to encounter the higher-value substrate and thus leading to more eggs on the higher-value option.

**Fig. 5:**
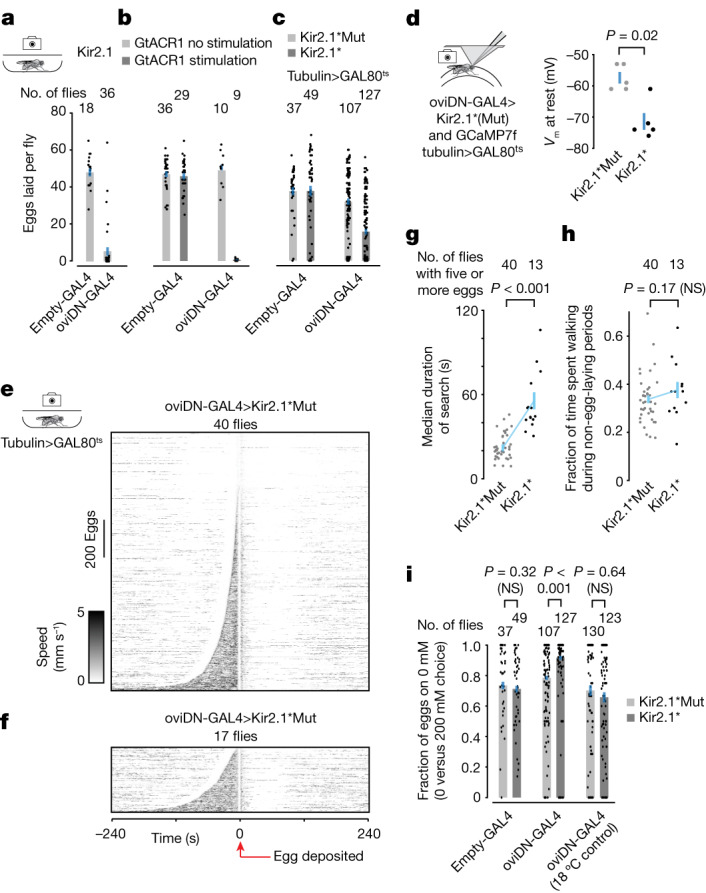
Gentle hyperpolarization of oviDNs increases search duration and results in more eggs laid on the preferred option. **a**–**c**, Eggs laid per fly (mean ± s.e.m.). Each dot represents one fly. Inhibition of oviDNs with Kir2.1 (**a**), GtACR1 (**b**), or Kir2.1* (**c**). **d**, oviDN (or oviDN-like neuron) *V*_m_ at rest (mean ± s.e.m.). Five cells in five flies and five cells in four flies, respectively. *P* value was calculated using two-sided Wilcoxon rank-sum test. **e**,**f**, oviDN-GAL4>Kir2.1*Mut (**e**) and oviDN-GAL4>Kir2.1* (**f**) flies. Each row represents a single egg-laying event in a 0 versus 200 mM sucrose chamber, aligned to egg deposition, with the fly’s speed indicated by intensity of black shading. Rows ordered based on the search duration; 1,377 eggs from 40 flies (45 flies tested, of which five did not lay eggs) and 346 eggs from 17 flies (40 flies tested, of which 23 did not lay eggs), respectively. **g**, Median duration of search for individual flies from **e**,**f** that laid five or more eggs. Mean ± s.e.m., *P* = 9.6 × 10^–7^. **h**, Fraction of time spent walking during non-egg-laying periods for flies shown in **g**. Non-egg-laying periods were defined as periods of over 10 min from egg deposition. **i**, Fraction of eggs on the lower-sucrose option with 95% confidence interval. Each dot represents one fly. Individual flies laid an average of 38, 38, 32, 16, six and seven eggs each. If the plot is reworked by examining only flies that laid at least five eggs, *P* = 1.9 × 10^–6^ (rather than 6.3 × 10^–4^) for the middle set of bars and is not significant (NS) for the others. **g**–**i**, *P* values calculated using two-sided Wilcoxon rank-sum test. **c**–**i**, Tubulin>GAL80^ts^ was present in all flies, to limit the time window in which Kir2.1* or Kir2.1*Mut transgenes were expressed (Methods). The 18 °C control was not shifted to 31 °C before the assay and thus expression of Kir2.1* or Kir2.1*Mut was not induced. All egg-laying experiments were conducted at 24 °C.

**Extended Data Fig. 10:**
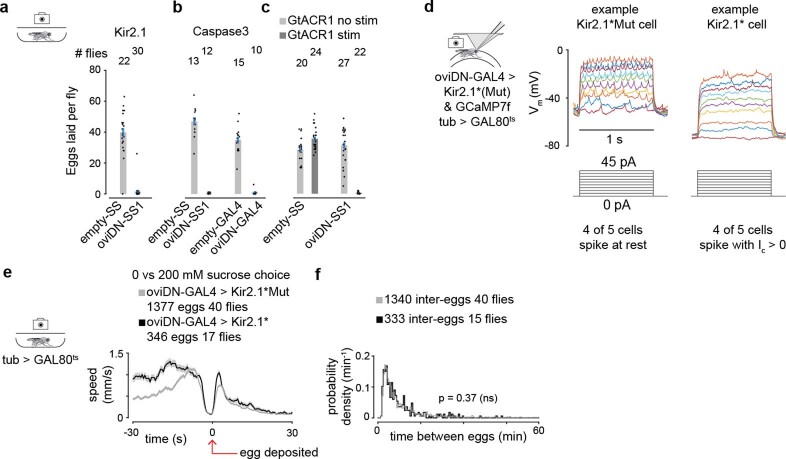
Strong and gentle inhibition of oviDNs. **a-c**, Eggs laid per fly. Each dot is one fly. ± s.e.m. indicated. **d**, The V*m* of a single, representative oviDN (or oviDN-like neuron) expressing Kir2.1*Mut or Kir2.1* during current injection. Four out of five Kir2.1* expressing cells showed spikes with sufficient amounts of current injection; one cell did not (not shown). **e**, Mean locomotor speed aligned to egg deposition with ± s.e.m. shaded. A higher average speed before egg laying in oviDN>Kir2.1* flies is indicative of the longer search duration in these flies. However, other aspects like the pause to lay an egg and post-egg-laying speed remain similar in oviDN>Kir2.1*Mut and oviDN>Kir2.1* flies. 1377 eggs from 40 flies (45 flies tested and 5 laid no eggs), 346 eggs from 17 flies (40 flies tested and 23 laid no eggs) for oviDN>Kir2.1*Mut and oviDN>Kir2.1*, respectively. **f**, Normalized inter-egg interval histograms. 1340 intervals from 40 oviDN>Kir2.1*Mut flies (45 flies tested and 5 laid < 2 eggs and thus did not have at least one interval). 333 intervals from 15 oviDN>Kir2.1* flies (40 flies tested and 25 flies laid < 2 eggs and thus did not have at least one interval). Note that the similar inter-egg interval distribution for oviDN>Kir2.1* and control flies does not mean that oviDN>Kir2.1* flies searched for the same amount of time for an egg-laying substrate as controls; rather, oviDN>Kir2.1* flies searched longer than controls (Fig. 5g). What is going on, remarkably, is that oviDN>Kir2.1* flies perform their next ovulation sooner after laying an egg than controls, such that despite searching longer before laying an egg, these flies ended up expressing nearly identical inter-ovulation and inter-egg intervals as control flies. The inter-ovulation interval (as estimated with locomotor speed) was not statistically different in oviDN>Kir2.1* and control flies (P = 0.36) (data not shown). P-values were calculated using two-sided Wilcoxon rank sum test.

Expressing the human Kir2.1 (ref. ^23^) potassium channel in oviDNs completely eliminated egg laying^9^ (Fig. 5a and Extended Data Fig. 10a), as did genetic ablation of oviDNs^9^ (Extended Data Fig. 10b) and optogenetic inhibition using the light-gated anion channel, GtACR1 (ref. ^24^) (Fig. 5b and Extended Data Fig. 10c). Each of these perturbations probably prevented the decision process from ever reaching threshold. Serendipitously, however, we introduced a modified mouse Kir2.1 (hereafter Kir2.1*) and a non-conducting control (Kir2.1*Mut) channel into *Drosophila*^25^ and found that flies expressing Kir2.1* in all oviDNs (oviDN>Kir2.1* flies) could still lay eggs, albeit at lower mean levels compared with genetic-background-matched controls (Fig. 5c and Methods). Whole-cell, patch-clamp recordings showed that Kir2.1*-expressing oviDNs (or oviDN-like neurons) were hyperpolarized by around 14 mV, on average, compared with Kir2.1*Mut-expressing (control) cells (Fig. 5d). This is a moderate hyperpolarization that still permitted most Kir2.1*-expressing neurons to fire spikes with sufficient current injection (Extended Data Fig. 10d). This fact could explain why many oviDN>Kir2.1* flies could lay eggs.

We tracked the *x*–*y* trajectories and egg-laying behaviour of oviDN>Kir2.1* and oviDN>Kir2.1*Mut flies in two-substrate, free-behaviour chambers. We observed a two- to threefold increase in the length of the search period in oviDN>Kir2.1* compared with oviDN>Kir2.1*Mut flies when comparing the full distribution of traces from all flies (*P* < 0.001; Fig. 5e,f and Methods), or when quantifying median search duration per fly (comparing flies that laid sufficient eggs for analysis—that is, at least five eggs; Fig. 5g). The increase in search duration could not be attributed to a general increase in the fraction of time spent walking (Fig. 5h), nor to a broad defect in egg-laying-related motor functions (Extended Data Fig. 10e,f). Remarkably, just as we imagined, the increase in search duration was accompanied by a higher fraction of eggs laid on the substrate of higher relative value (Fig. 5i), probably because oviDN>Kir2.1* flies have more time to encounter the higher-relative-value option before threshold is reached.

## A neural circuit for egg laying

**Fig. 6:**
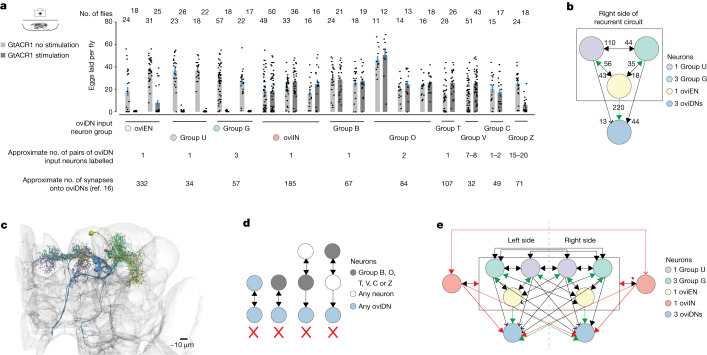
An anatomically recurrent neuronal circuit whose activity is required for egg laying provides direct synaptic input to oviDNs. **a**, Eggs laid per fly (mean ± s.e.m.). Each dot represents one fly and each pair of bars represents a split-GAL4 line (Supplementary Table 1). Estimate of number of pairs of oviDN input neurons and number of synapses onto oviDNs is explained in Methods. Labelling of oviENs in second split-GAL4 is stochastic (Extended Data Fig. 11b), explaining why some flies still lay eggs. **b**, Hemibrain-derived connectivity of indicated neurons on one side of the brain. Numbers adjacent to arrows indicate total synapse counts. Green arrows indicate excitatory (oviENs are cholinergic^9^); black arrows are of unknown sign but are posited to be excitatory. Arrows drawn only if connection has more than two synapses. Arrows with filled arrowheads indicate that there exists a single neuron–single neuron connection with at least ten synapses. **c**, Recurrent-circuit neurons on the right side of the brain using Neuroglancer and the hemibrain connectome. **d**, Hemibrain-derived connectivity of indicated neurons on either side of the brain. Filled arrows indicate a single neuron–single neuron connection with at least ten synapses. X indicates that the diagrammed connection does not exist at a threshold of ten or more synapses. **e**, Hemibrain-derived connectivity. Green and black arrows are as in **b**, and red arrows are inhibitory (oviINs are GABAergic^9^); arrows with filled arrowheads are as in **b** (see Supplementary Tables 3 and 4 for all synapse counts). Light blue circles represent three oviDNs on the right side and one on the left. Only one oviDN on the left side of the brain is annotated in the hemibrain, and was used to capture connectivity on that side. OviINs receive input from, and send output to, each individual neuron within the box. Arrow marked by * indicates that no individual group G (right) synapses onto oviIN (right) with ten or more synapses.

**Extended Data Fig. 11:**
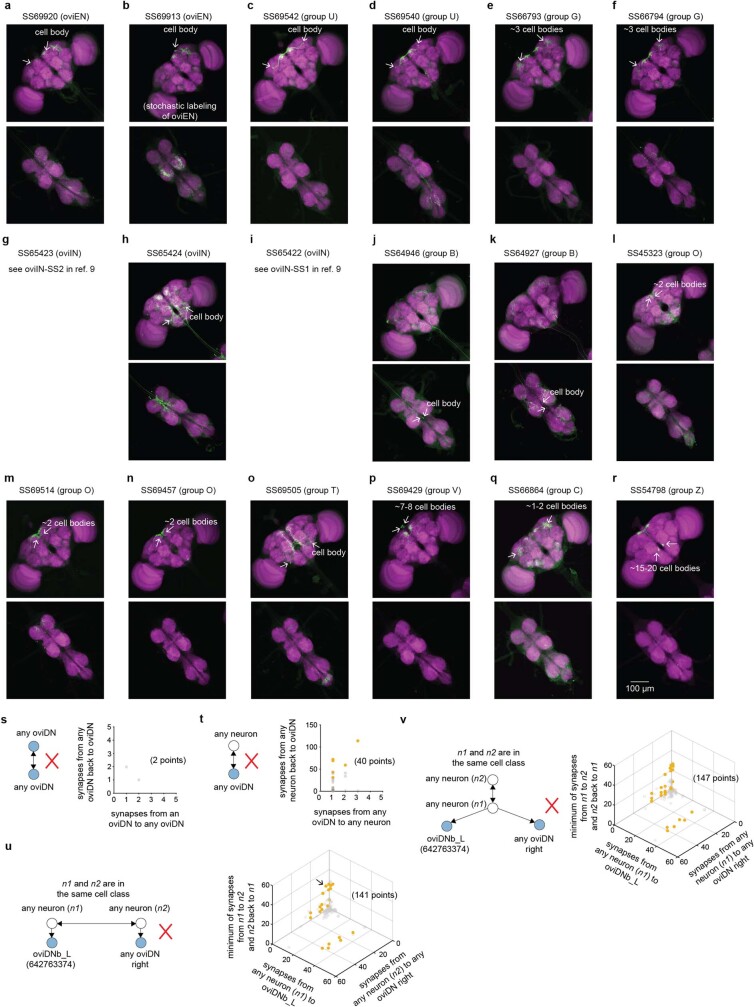
Spilt-GAL4 lines targeting oviDN input neurons and analysis of oviDN inputs in the hemibrain connectome. **a-r**, Average z-projection of oviDN input split-GAL4 lines in the brain (top) and ventral nerve cord (bottom) in order of x-axis in Fig. 6a (see Supplementary Table 3 for additional information). Green shows UAS-CsChrimson-mVenus expression in the targeted neurons and magenta represents a neuropil counterstain (Methods). **s**, In panels s-v, we show circuit motifs that are not supported by our analysis of the hemibrain connectome^16^, in contrast to the motif reported in Fig. 6e, which is supported (see Methods for more discussion). This panel shows that no chemical-synapse-based recurrent circuit is observed between the oviDNs themselves in the hemibrain connectome at a threshold of ≥ 10 synapses per connection (even true at a threshold of ≥ 2 synapses here). (For reference on the potential functional significance of a 10 synapse threshold, ~15-20 neurons make ≥ 10 synapses onto an individual oviDN in the hemibrain.) Scatter plot of connections between pairs of neurons is shown to right. Both data points are from a single pair of oviDNs. **t**, We found in the hemibrain connectome all pairs of cells that fulfilled the circuit diagram shown on the left at a threshold of ≥ 1 synapse per connection. No direct, bidirectional, chemical-synapse-based recurrent circuit could be detected between individual oviDNs and any oviDN input neuron in the hemibrain connectome at a threshold of ≥ 10 synapses per connection (even true at a threshold of ≥ 4 synapses per connection here). Scatter plot of connections shown to right; each dot represents the connections between two neurons. Orange points represent pairs of connected neurons diagrammed in the recurrent circuit in Fig. 6e, but assayed for participation in a different circuit motif here. **u**, We found in the hemibrain connectome all sets of four cells that fulfilled the circuit diagram shown on the left at a threshold of ≥ 1 synapse per connection. None of these putative circuits had ≥ 10 synapses for all four connections, which we interpret to mean that no chemical-synapse-based, disynaptic recurrent circuit exists between individual oviDNs and a single class of oviDN input neuron. Cell classes (types) were based on the hemibrain v1.2.1 connectome annotation^16^. Scatter plot of connections is shown to right; each dot represents the connections between a set of four neurons. Orange points represent sets of four neurons diagrammed in the recurrent circuit in Fig. 6e, but assayed for participation in a different circuit motif here. For example, the orange dot indicated by an arrow represents the following connections between single cells: oviDNa(right)←groupU(right)↔groupU(left)→oviDNb(left). **v**, Same as panel u, but for a different circuit architecture. Each point, once again, represents the connections between a set of four neurons. None of these putative circuits had ≥ 10 synapses for all four connections.

Finally, we wished to provide an inroad into the circuit mechanisms underlying the rising [Ca^2+^] signal in oviDNs. We created split-GAL4 driver lines that allowed selective inhibition of several neuron classes that have extensive synaptic input onto oviDNs^16^ (Methods, Supplementary Table 3 and Extended Data Fig. 11a–r). We found three groups of neurons—oviEN^9^, group U cells and group G cells—that when inhibited with GtACR1 markedly reduced the total number of eggs laid by flies (Fig. 6a; see Methods for discussion of group Z). Although oviEN activity is known to be required for egg laying^9^, the requirement for activity in group U and group G neurons—which make far fewer direct synapses onto oviDNs than oviENs or many of the other neuron types tested (Fig. 6a)—is a new finding.

**Extended Data Fig. 12:**
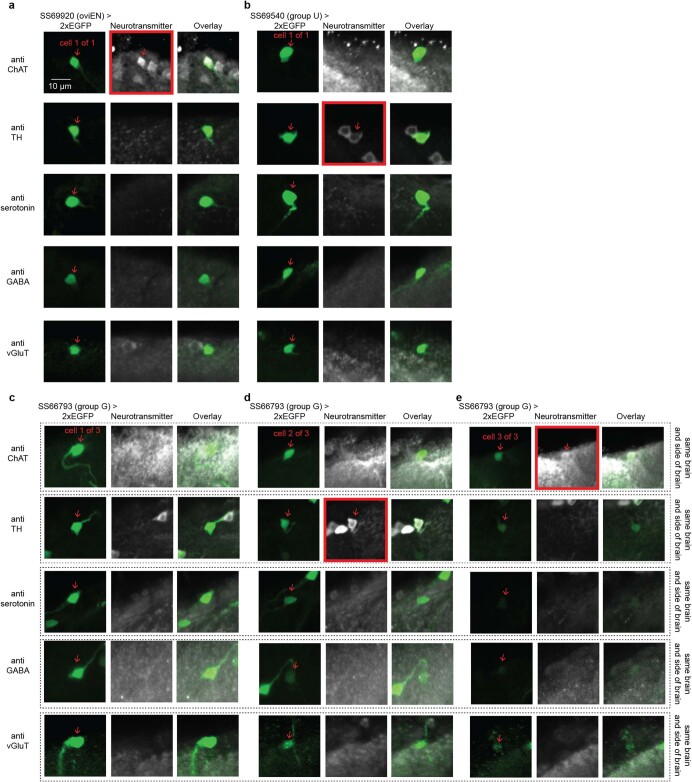
Neurotransmitter identity of recurrently connected neurons. **a–e**, Average z-projection of 5 z-slices (1 µm z-intervals) centered around the cell of interest. oviEN cells are ChAT positive (panel a); group U cells are TH positive (panel b); and the staining of the 3 group G cells (assigned to cell 1, cell 2, or cell 3 with decreasing brightness of 2xEGFP immunostaining) yielded one cell with unclear transmitter assignment (panel c, cell 1 of 3), one TH positive cell (panel d, cell 2 of 3), and one ChAT positive cell (panel e, cell 3 of 3), respectively. The three group G cells consistently had three qualitatively different levels of brightness (2xEGFP in panels c-e).

To identify what might be special about oviEN, group U, and group G cells we analysed their connectivity in the hemibrain^16^, discovering that these cells, at the anatomical level, form a recurrent circuit that feeds into oviDNs (Fig. 6b,c and Supplementary Table 4). This recurrent circuit comprises just five neurons per side of the brain, and silencing any of its constituent neuron groups eliminates egg laying, presumably by preventing the decision process from ever reaching threshold. None of the other groups of neurons we tested formed a recurrent circuit with the same or fewer number of neurons (Fig. 6d; see Methods for further analysis and discussion; Extended Data Figs. 11s–v and 12). Cells in this circuit on both sides of the brain are reciprocally connected, and a pair of GABAergic inhibitory neurons, oviINs^9^, may act to keep activity in the circuit from rising too rapidly, in addition to gating egg laying on the basis of internal state^9^ (Fig. 6e).

## Discussion

Rise-to-threshold signals have been linked to decision-making and action-initiation processes in humans^26^, monkeys^3,27–30^, rodents^31–34^, zebrafish^35–37^ and insects^38–42^. These signals have been shown to rise, or suggested to rise, on the hundreds-of-milliseconds to seconds time scale. Some of the most influential work in this domain has focused on rise-to-threshold signals that integrate noisy sensory input so that an animal can report a percept^1–3^—that is, form a ‘perceptual decision’. Our work helps to extend the rise-to-threshold framework beyond perceptual decisions to ethologically relevant, self-paced decisions in which animals decide among non-noisy, perceptually distinct, options^43^ (for example, egg-laying substrates with easily distinguishable differences in sucrose concentrations). Our work further emphasizes three features of rise-to-threshold processes that were not easily appreciated previously: (1) they can regulate decisions that take minutes, not just seconds; (2) they can cause behaviour to start when threshold is crossed^33,41^; and (3) their rate of rise can be modulated by the relative value (and not just the more veridical sensory properties) of stimuli. These features expand on past work on rise-to-threshold processes^26–42^, suggesting that they may underlie a wide array of ethological, self-paced decisions made by animals in the real world.

Recurrent neural circuits have been proposed as a mechanism for rising or persistent neuronal activity^44,45^. Here we describe a small, anatomically recurrent circuit where silencing activity in any constituent cell class eliminates egg laying. Although we have not yet measured physiological activity in all circuit constituents during egg laying, we speculate that synaptic interactions in this circuit contribute to the generation of a rising or persistent oviDN spike rate, which is then integrated by oviDN’s slow calcium dynamics to create the signal we report in this paper.

If one compares a fly’s decision to lay an egg in an environment with several discrete substrate options^20^ with a human’s decision to choose a dish at a restaurant, there are interesting parallels. Both processes start with an initiation event: ovulation in flies or opening a menu in humans. Then the individual’s own behaviour reveals new options over time—that is, more egg-laying substrates to the fly walking around an environment or more dish options to the human scanning the menu. Finally, the decision is terminated when one option is selected and a motor programme, of varying complexity and delay relative to the end of the decision, is implemented. This analogy highlights that the process characterized herein may help to inform decision-making quite broadly.

## Methods

### Flies

Flies (*D. melanogaster*) were reared on a standard cornmeal medium at 25 °C, ambient humidity and 12/12 h light/dark cycle unless otherwise noted. Genotypes and conditions for each experiment are described in Supplementary Tables 1 and 2, respectively. Supplementary Table 1 also lists the source of each genotype.

### Egg-laying chamber with sloped ceiling

We designed a new chamber for imaging egg laying in freely walking flies, which enforced them to remain in a tarsi-down body posture on the agarose at all times. The flies could not tilt their bodies in this chamber and thus they could not walk on the side walls or ceiling. This constraint meant that the flies’ bodies were always in the same general orientation, parallel to the imaging plane, making quantitative measurements of postural parameters more straightforward with a single camera view.

Chambers were made by sandwiching and tightly screwing layers of acrylic and three-dimensionally (3D)-printed plastic and then fitting a glass ceiling (Extended Data Fig. 1a). The acrylic layers were laser-cut (VLS6.60, Universal Laser Systems). The side-wall layer was 3D-printed using VisiJet M3 Crystal plastic material (Projet 3510 HD Plus, 3D Systems). The glass was treated with Sigmacote (Sigma-Aldrich) to make it slippery to a fly’s tarsi—preventing walking on the ceiling^46^. Glass was retreated with Sigmacote after roughly ten uses. The 3D-printed spacer layer incorporated a sloped edge that kept the fly completely parallel to the imaging plane by preventing access to the side of the chamber (Extended Data Fig. 1a). The sloped-ceiling design was inspired by a sloped-floor plastic chamber^46^. A sloped floor does allow the fly to tilt and thus was not suitable for our application.

Chambers were used multiple times and washed before each use. They were assembled with only the two bottom layers and then cooled at 4 °C. Fresh substrate containing 1% agarose (SeaKem LE Agarose, Lonza), 0.8% acetic acid and 1.6% ethanol was pipetted to completely fill the well around 5 h before each assay. Careful pipetting with only the two bottom layers assembled was critical to forming a flat layer of agarose—preventing the formation of a meniscus, which would allow the fly to tilt. Acetic acid and ethanol were included to help simulate rotten fruit and generally promote egg laying^7^. After solidification of the agarose solution (about 1 h) the chamber was fully assembled, minus the glass ceiling, and equilibrated at room temperature.

Females were separated on their day of eclosion and group housed in vials. At age 3–6 days around 20 females were exposed to about 20 Canton-S males in an empty bottle with wet yeast paste and a Kimwipe (Kimberly-Clark) soaked with 2 ml of water. The wet yeast paste was applied to the side of the bottle and comprised 1 g of dry yeast (Fleischmann’s) and 1.5 ml of 4.25 mM putrescine dihydrochloride in water. This treatment allowed females to mate and caused them to accumulate many eggs. Flies fed with yeast^7,47^ or putricine^48^ increase the number of eggs they develop. These eggs are retained by the flies during the treatment period because they lack a soft medium for egg deposition^7^. After about 24 h, individual gravid females were placed into chambers under gentle cold anaesthesia from which they typically recovered within 30 s. Because we had only one imaging setup for these high-resolution experiments (see below), and the ability of a fly to tilt was sensitive to both its size the exact level of agarose, multiple flies were loaded in independent chambers (Extended Data Fig. 1a) and the fly with the least ability to tilt was chosen for imaging for a few hours in near-complete darkness (under a shroud) at around 24 °C and 40–60% humidity.

For imaging eggs inside the fly’s body, a 470 nm LED (pE-100, CoolLED) double filtered (optical density (OD) 4 475 nm and OD 4 500 nm shortpass, Edumund Optics) provided excitation light at 30 µW mm^–1^. This excitation light arrived at the fly from below after first passing through the agarose substrate. Videos were recorded using HCImage software (Hamamatsu) at ten frames s^–1^ (fps) with 100 ms exposure time per frame, using an ORCA-Fusion C14440-20UP camera (Hamamatsu) equipped with a 15.5–20.4 mm Varifocal Lens (Computar) and two 510 nm longpass filters (Chroma). We used GCaMP3, rather than the more recent GCaMP variant, for imaging of eggs because a UASp-driven GCaMP3 transgene, which is more highly expressed in the female germline than the traditional UAS^49^, was constructed in a previous study^15^ and available for use without the need to generate a new transgenic fly.

For imaging of body posture, 850 nm LEDs illuminated the arena from above through a white acrylic diffuser (1 µW mm^–2^ at the fly). Videos were recorded at 25 fps using FlyCapture software (FLIR) and a GS3-U3-41C6NIR-C Grasshopper camera (FLIR) equipped with a 15.5–20.4 mm varifocal lens and a 780 nm longpass filter (MidOpt). DeepLabCut^50^ was used for offline tracking of body parts, including the neck and ovipositor. DeepLabCut models were iteratively fine-tuned by identification of poorly tracked frames in iteration *i* and adding them to the training dataset for iteration *i* + 1. A total of 1,568 training frames were manually annotated. DeepLabCut output coordinates were filtered by setting coordinates to not-a-number (NaN) if either (1) the probability score was less than 0.95 or (2) the body part jumped more than an empirically determined distance in consecutive frames. Ovulation start was manually annotated as the first frame in which the abdomen appeared to begin the elongation process. Search start was manually annotated as the first frame in which the abdomen returned to a stable neutral posture after ovulation. Abdomen bend complete was manually annotated as the frame in which the bend to lay an egg was completed (abdomen maximally deflected). Identification of the frame in which the abdomen bend was completed was much easier than attempting to identify when the abdomen bend was initiated. Note that, although flies bend their abdomen to deposit an egg, they also bend their abdomen for other reasons. Some non-egg-laying-related reasons a fly could bend its abdomen include defaecation, grooming and sampling the substrate with sensory organs near the ovipositor. ‘Egg deposited’ was manually annotated, often with assistance from a computer algorithm. Briefly, our computer code found groups of pixels whose intensities stably changed at a particular frame in the video. The output frame numbers from the code pointed an experimenter to video frames proximal to egg deposition, and the exact frame for egg deposition was adjusted manually. Videos were also carefully inspected by an experimenter to identify eggs missed by the code. This code markedly accelerated manual annotation and was particularly useful for high-throughput egg-laying choice chambers where thousands of eggs were annotated (see below). The first frame in which half of the egg was visible (emerging from the ovipositor) was annotated as the egg-deposited frame.

### High-throughput egg-laying choice chamber

We designed a new chamber for studying egg-laying choice behaviour with high throughput. This chamber ensured that the fly was nearly always in contact with an agarose egg-laying substrate option. The substrate on which the fly was standing could be unambiguously defined by its *y* position and orientation. In previous egg-laying choice studies^8,51,52^, flies could walk on the side walls or ceiling and yet were assigned to a substrate beneath them during tracking, which makes it very hard to determine how previous substrate experiences influence the decision to lay an egg.

Chambers were made by sandwiching and tightly screwing layers of acrylic or Delrin plastic and then affixing a glass ceiling (Extended Data Fig. 1b). Acrylic and Delrin plastic were laser-cut and the glass was treated with Sigmacote.

Chambers were used multiple times and washed before each use. They were assembled without the glass ceiling and cooled at 4 °C. Fresh substrate (1 ml, containing 1% agarose, 0.8% acetic acid and 1.6% ethanol) was pipetted to fill the acrylic well and form a meniscus with the Delrin plastic spacer about 5 h before each assay. The meniscus ensured that the fly could not walk directly on the side (Delrin plastic) of the chamber and was inspired by plastic chambers with a sloped floor^46^. Quantitative measurements of body posture were not possible because flies could tilt by walking on the meniscus. Sucrose-containing substrates were supplemented with the appropriate amount of sucrose. Acetic acid and ethanol were uniformly distributed in all substrates. Following solidification of the agarose solution (about 1 h), the chamber was equilibrated at room temperature.

These egg-laying chambers and assay protocols were specifically designed to minimize the following confounds: (1) diffusion between substrate islands; (2) visual landmarks; (3) fly-to-fly communication; (4) olfactory landmarks; (5) temperature and humidity fluctuations; and (6) variability in fly rearing. Diffusion was minimized by a barrier of approximate width 2.5 mm between the substrate islands and by loading the agarose at 4 °C. Visual cues were minimized by conducting the assay in near-complete darkness. Illumination of 850 nm, to which the fly’s visual system has no measurable sensitivity^53–55^, was provided from below for tracking (1 µW mm^–2^ at the agarose beneath the fly). Fly-to-fly communication was minimized by assaying individual flies in isolated chambers separated by an opaque Delrin plastic spacer. Olfactory landmarks were minimized using a non-volatile compound, sucrose, as the sole varying variable. Temperature and humidity were kept constant by conducting experiments in an environmental room (24 °C with 40–60% humidity). Air exchange was made possible by four small ventilation holes in each barrier. Variability in fly rearing was minimized by controlling age, mating status, food history and circadian time.

Females and males were separated on their day of eclosion and group housed in vials. At age 3–6 days at zeitgeber time (ZT) 6 (that is, 6 h after lights on), around 20 females were exposed to around 20 Canton-S males in an empty bottle with only wet yeast paste and a Kimwipe soaked with 2 ml of water. Putrescine was not added to the yeast paste in these experiments. On the following day at ZT 8, individual females were placed into egg-laying chambers under gentle cold anaesthesia. Videos were acquired at 2 fps using FlyCapture software with either a FMVU-03MTM-CS Firefly or FL3-U3-13Y3M-C Flea3 camera (FLIR) equipped with either a LM12HC (Kowa), HF12.5SA-1 (Fujinon) or CF12.5HA-1 (Fujinon) lens and a 780 nm longpass filter. The *x–y* position and orientation of each fly was determined offline using Ctrax^56^. We assigned a fly to a substrate depending on whether its centroid was above or below the midline of the acrylic barrier. This simplification was appropriate because the acrylic barrier of roughly 2.5 mm (a fly is around 2.5 mm long) practically prevented a fly from standing on both substrates simultaneously, and a Canton-S fly spent only 1.5% of its time in an orientation where all tarsi were likely to be on the barrier. Note that flies do not lay eggs on the plastic barrier (or any plastic used in this study) because it is too hard. Egg deposition was manually annotated, often with the assistance of a computer algorithm, as described in the previous section. The first frame in which half of the egg was visible (emerging from the ovipositor) was annotated as the egg-deposited frame. Annotations by an individual human annotator or across multiple human annotators were reproducible to ±four frames or ±2 s.

For Kir2.1* or Kir2.1*Mut experiments we expressed a GAL80^ts^ transgene in all cells (with the tubulin promoter)^57^ during development to minimize transcription of Kir transgenes days before assaying egg-laying behaviour. At 18 °C, GAL80^ts^ masks the transcription activation domain of GAL4, thus preventing transcription of the GAL4-UAS-controlled transgene. We could remove the GAL80 block on Kir expression by increasing the flies’ temperature for about 1 day before our egg-laying assays. Specifically, for these experiments: (1) flies were reared at 18 °C; (2) at ZT 6 flies were moved to 31 °C for induction of Kir2.1* or Kir2.1*Mut transgene expression; and (3) the following day at ZT 5 (23 h later), flies were returned to 18 °C. Egg-laying assays were performed at ZT 8 at 24 °C. For one set of controls in Fig. 5i, flies were not moved to 31 °C and instead were kept at 18 °C.

For GtACR1 (refs. ^24,58^) experiments, flies were kept under low white light (approximately 3 nW mm^–2^ measured at 567 nm) from egg to adulthood. At approximate age 5–6 days at ZT 6, around ten females were exposed to around ten Canton-S males in an empty bottle with only wet yeast paste and a Kimwipe soaked with 2 ml of 200 µM all-transretinal in water (also kept under low white light). Wet yeast paste was applied to the side of the bottle and comprised 1 g of dry yeast with 1.5 ml of 200 µM all-transretinal in water. Egg-laying assays were performed the following day at ZT 8. Light (567 nm) was provided from above (29 µW mm^–2^ at the fly; Rebel Tri Star LEDs, LuxeonStarLEDs). Controls for genotype were siblings of experimental flies that were treated identically except that no light was provided from above. Controls for light were flies ‘expressing’ GtACR1 with either an empty-split (empty-SS) or empty-GAL4 driver. Additional controls for light with twice the intensity (57 µW mm^–2^) provided additional assurance that light alone was not preventing egg laying (data not shown).

### Construction of Kir2.1* and Kir2.1*Mut flies

We serendipitously identified that Kir2.1*^25^ (based on the mouse sequence for the gene, see below) hyperpolarizes oviDNs more gently than the human Kir2.1 traditionally used in flies^23,59,60^ (Fig. 5c versus Fig. 5a). A matched control channel, Kir2.1*Mut^25^, does not conduct ions and enabled genetic-background-matched comparisons. A similar strategy of using Kir2.1 paired with a non-conducting control was recently used in flies^61^, although with the human variant of the gene.

Kir2.1* and Kir2.1*Mut sequences were taken from a previous study in mice^25^. Briefly, Kir2.1* and Kir2.1*Mut are modified wild-type mouse Kir2.1 channels (KCNJ2)—with either two mutations (Kir2.1*: E224G, Y242F) or five mutations (Kir2.1*Mut: E224G, Y242F, G144A, Y145A, G146A). Both transgenes were fused at their C-terminals with a T2A sequence to a tdTomato. To port these constructs into *Drosophila*, they were inserted between the Xba1 and Not1 sites of pJFRC81 (ref. ^62^) and introduced into the *attP40* landing site by ΦC31 integrase-mediated transgenesis (transgenic fly lines were generated by BestGene). Kir2.1* and Kir2.1*Mut transgenes differ in protein sequence—and possibly in other ways (for example, transcription and translation)—from the wild-type human Kir2.1 (KCNJ2) transgenes traditionally used to hyperpolarize neurons in flies^23,59,60^. Previous in vivo fly electrophysiology of central brain and visual system neurons expressing traditional human Kir2.1 (refs. ^63,64^) transgenes showed larger hyperpolarization than the approximately 14 mV hyperpolarization observed here with Kir2.1* (Fig. 5d).

### Automated estimation of search period in free-behaviour, high-throughput choice chambers

Because we did not have a quantifiable view of the abdomen in our high-throughput choice chambers (Extended Data Fig. 1b,c), we used locomotor speed as a proxy for search onset (Extended Data Fig. 1d–f) and egg deposition as a proxy for abdomen bending to lay an egg (Fig. 1c). The end of the search period was the annotated moment of egg deposition (rather than the abdomen bend to lay the egg). For each egg, the start of the search period was determined by smoothing the locomotor speed trace before egg deposition with an 18.5 s boxcar filter and identifying the first frame in which the smoothed signal fell below 0.1 mm s^–1^. Due to the length of the boxcar filter, the minimum search duration was 9 s. These parameters were empirically established to produce search onset times that were consistent with what an expert human annotator would highlight in visual analysis of the data.

### Calculation of egg-laying rates as a function of time since the last substrate transition in free-behaviour choice chambers

Egg-laying rates as a function of time (Fig. 3f–h) were calculated as follows. Before performing any calculations, we combined the data obtained from all flies tested in a particular chamber type. First, we iterated through each time bin on the *x* axis and, for each bin, we counted the number of egg-deposition events assigned to that bin, denoted as #_eggs_(bin). Next, we repeated the iteration for the same time bins and tallied the number of video frames in which the flies were assigned to that time bin, referred to as #_frames_(bin), during a search period. Finally, we performed another iteration for the same time bins and recorded the number of times flies changed assignments into that bin, termed #_visits_(bin), during an egg-laying search period (that is, we didn't keep incrementing the ‘visits’ counter if the fly remained in a particular time bin from one frame to the next).

To determine the mean egg-laying rate, we computed #_eggs_/#_frames_ for each bin. Because the videos were recorded at 2 Hz, we multiplied the value obtained for each bin by 120 to convert it to units of eggs min^–1^. To determine the confidence interval for each bin we utilized the Clopper–Pearson method, also known as the ‘exact’ binomial confidence interval, to compute the 90% confidence interval for #_eggs_/#_visits_. We then transformed the confidence interval for each bin to units of eggs min^–1^ by multiplying it by 120 × #_visits_/#_frames_. The confidence interval could not be directly calculated from #_eggs_/#_frames_ because it would then be contingent on the video frame rate.

For these rate curve calculations, search periods with duration shorter than 30 s were set to 30 s. This prevented very brief search periods from introducing fluctuations in the rate functions (by contributing to the numerator and not contributing much to the denominator). By doing so, the rate curves exhibited less variation across replicates or conditions. Note that search periods already had a minimum duration of 9 s, which was automatically determined by the search period calculation (Methods). Altering the definition of the search period, or having no minimum search duration, does not change our stated conclusions from these curves^20^. Additionally, the use of different *x*-axis bins yields qualitatively similar results and does not change our stated conclusions. Rate functions start with low rates after a transition, at least partially, because flies do not lay eggs on the plastic barrier between substrates (Extended Data Fig. 7e,f) and because flies are, by definition, walking (and not pausing to deposit an egg) during a transition (Extended Data Fig. 7g).

### Design of egg-laying wheel and setup under microscope

We designed a wheel on which tethered flies walked and laid eggs on agarose-based egg-laying substrates. The design was optimized to maximize a fly’s ability to lay eggs and rotate the wheel.

The wheels were 3D printed from VisiJet M3 Crystal plastic using a Projet 3510 HD Plus 3D printer (Extended Data Fig. 2a). A pivot (N-1D, Swiss Jewel) was press-fit through the centre hole and not removed. Wheels were washed before each use. Three wells were available for loading the same or different agarose-based substrates. Each well was separated by a 1 mm barrier. Wheels were loaded with fresh agarose substrate (as prepared for free-behaviour choice chambers) using a 3D-printed agarose-injecting mould (VisiJet M3 Crystal material) that was cooled on ice (Extended Data Fig. 2b). Food colouring (HY-TOP assorted food colouring) was added at a dilution of 1:10,000 to the agarose solution before loading so that wheel quality could be visualized. Wheels with any mixing between wells were discarded. Food colouring at 2.5-fold this concentration, or the presence of VisiJet M3 Crystal material, did not affect choice in free-behaviour control experiments (Extended Data Fig. 2d). After solidification of the agarose was, the wheel and pivot were suspended between two spring-loaded bearings (VS-30, Swiss Jewel) threaded into clear acrylic that was press-fit into a 3D-printed base (UMA-90 material printed on a Carbon DLS, Protolabs) (Extended Data Fig. 2c). This wheel assembly was stored in a custom humidification chamber to prevent the thin layer of agarose from drying and to allow the wheels to equilibrate to room temperature. Wheels were used within 2 h of preparation. When ready, a wheel assembly was secured in a small custom humidification chamber (roughly 90% humidity) positioned under the microscope objective. The wheel–pivot combinations used in this study had a weight of 87.9 ± 0.3 mg (mean ± s.d.) without agarose and 146.4 ± 0.8 mg with agarose. For reference, a single gravid female weighs around 1.4 mg and a typical foam ball used for fly walking experiments^65,66^ weighs 40–46 mg. Most of the wheel’s weight is due to the agarose and the wells needed to hold it. A variety of lighter and synthetic materials less prone to evaporation were screened in free-behaviour assays, but egg laying was suppressed in all of them.

The fly was viewed using two CM3-U3-13Y3M Chameleon cameras (from the sides) and one FMVU-03MTM-CS Firefly camera (FLIR) from the front, and videos were captured using FlyCapture software. Two 850 nm LEDs, from front left and front right, illuminated the fly at 5 µW mm^–2^. Cameras were equipped with a 15.5–20.4 mm varifocal lens and either a 900 nm shortpass (Thorlabs) or 875 nm shortpass (Edmund Optics) filter to dampen visibility of the 925 nm two-photon excitation light. Cameras had an exposure time of 16 ms and were triggered synchronously using a single external trigger source at 25 fps (Arduino Uno, Arduino). A side-facing camera recorded the fly and a single dot painted on the wheel. The dot was painted in a consistent location on the wheel that was defined by an embossed 3D-printed feature. The dot was tracked using DeepLabCut (1,109 training frames, with training and filtering as in the free-behaviour DeepLabCut model). The dot position was converted to wheel degrees by fitting the set of all dot positions to a circle and then computing a wheel angle for each frame. A single frame in which the fly’s centroid straddled the dot was used to convert the wheel angle to the fly’s position on the wheel. This alignment consistently meant that the fly’s neck was situated on the plastic-to-next-substrate boundary during a detected substrate transition. A second side-facing camera was used for a close-up view of the fly’s body. DeepLabCut was used to track body parts including the neck, ovipositor and tip of the proboscis (2,259 training frames, with training and filtering as in the free-behaviour DeepLabCut model). Normalized length was calculated by subtracting the *x*-coordinates of the neck and ovipositor in each frame and dividing by the median of this value for each recording (Fig. 1i). The median length in free behaviour was approximately 2.35 mm (Extended Data Fig. 1e–h), although we did not measure this value on the wheel. We used this normalized-length metric because it can quantify both an elongated and a bent abdomen and is similar to the neck–ovipositor length measured in free behaviour. Despite the similarity with free-behaviour length, we noticed, on average, a slight difference in the signature of abdomen bends (Extended Data Fig. 1g compared with Fig. 1l), possibly due to the curvature of the wheel. The body angle (°) was the angle between the neck and ovipositor (Fig. 1i). Larger angles indicated a more bent abdomen. Although a fly must bend its abdomen to lay an egg, the magnitude of a physiologically relevant deflection of body angle (as measured in degrees) is not that large (Fig. 1i). ‘Normalized neck to proboscis length’ was calculated by determining the Euclidean distance between the tip of the proboscis and the neck in each frame and dividing by the median of this value for each recording. This underestimated the true deflection of the proboscis because the proboscis does not start at the neck. The neck was used as an origin point because robust tracking was easy. A front-facing camera was used to align the fly on the centre of the wheel width. The body posture slightly varied among flies due to slight differences in tethering. To achieve egg laying it was very important to position the fly at a point on the wheel circumference, and at a vertical distance from wheel, that maximized perpendicular contact of the ovipositor to the substrate when the abdomen was bent while still allowing the fly to walk on the wheel. In some cases flies had to be positioned close to the wheel which, unfortunately, decreased the dynamic range of abdomen bending. A total of 104 flies were imaged to collect the data shown in Fig. 1h. The majority of flies did not lay eggs because, among other considerations, flies often require several hours to start laying their clutch of eggs (even in free behaviour). We could not image, conveniently, for 18 h to wait for a clutch to start.

Moments of distinct behaviours (as in Fig. 1h and Extended Data Fig. 2g) were annotated manually by inspection of behaviour videos while remaining blind to any neural signals (∆*F*/*F*). Ovulation start was defined as the first frame in which the abdomen appeared to begin the elongation process; ‘abdomen at its longest’ was the frame in which the abdomen was maximally stretched; ‘abdomen scrunch start’ was the first frame in which the abdomen assumed a stable scrunched position; search start was defined as the first frame in which the abdomen returned to a stable neutral posture after ovulation; abdomen bend complete was defined as the frame in which the first bend before egg laying was complete (abdomen maximally deflected); egg deposited was defined as the frame in which half of the egg was visible; and ‘ovipositor cleaned’ was defined as the frame in which the first abdomen bend following egg laying was complete.

For CsChrimson^18^ optogenetics experiments, a 660 nm LED coupled to a 1-mm-wide fibre-optic cable (M660F1 and M35L01, Thorlabs) was focused on the front midpoint of the fly’s head using a lens set (MAP10100100-A, Thorlabs). This wavelength is at the tail end of the sensitivity of the fly visual system^53–55^, which helps to minimize light-related confounds. Two longpass filters—OD 4 550 nm and OD 4 575 nm (Edmund Optics)—minimized the ability of LED light to enter the two-photon detector path, which collected the GCaMP signal. The incident area of the LED was adjusted to be of sufficient width (approximately 3 mm in diameter) to cover the whole front of the fly, from the part of the head glued to the custom holder to the tips of the tarsi (see Extended Data Fig. 2f for representative fly positioning), such that all CsChrimson-expressing oviDN cell bodies and neurites in the brain could be stimulated. CsChrimson-expressing oviDN neurites and synapses in the abdominal ganglion (situated in the thorax) were also probably stimulated—albeit to a lesser degree due to obstruction from the head, proboscis and front tarsi—because the whole front of the fly head and body was illuminated. LED intensity was controlled by adjusting the duty cycle of a 490 Hz PWM signal (Arduino Uno, Arduino) that was fed into an LED driver (T-Cube, Thorlabs). The CsChrimson stimulation intensity for Fig. 2a–e was 641 µW mm^–2^. For Fig. 2f–h and Extended Data Fig. 6a–f, intensities were 641, 159, 148 and 136 µW mm^2^. For the prolonged, gradual CsChrimson experiments in Extended Data Fig. 5o–s, data from three separate stimulation paradigms were combined: 159 µW mm^–2^ was applied (1) at 100 ms on, 400 ms off, for 30 s; (2) at 100 ms on, 900 ms off, for 39 s; or (3) at 50 ms on, 950 ms off, for 50 s. Sample traces shown in Extended Data Fig. 5o are both from stimulation paradigm (3). For Extended Data Fig. 6l–p, intensity was approximately 148 µW mm^–2^.

### Treatment of flies for tethered egg-laying and optogenetic experiments

Females and males were collected on their day of eclosion and group housed together in standard cornmeal medium vials supplemented with 2.5 mM putrescine dihydrochloride and wet yeast paste. Wet yeast paste was applied to the side of the vial and comprised 1 g of dry yeast and 1.5 ml of 4.25 mM putrescine dihydrochloride in water. At around age 5–6 days, females were gravid because larvae occupied the cornmeal medium and there was no additional room to deposit eggs. This treatment was more convenient than that used in free-behaviour choice experiments and was inspired by separate aspects of two studies^8,48^. Free-behaviour controls indicated that this treatment increased the number of eggs laid by a fly without affecting choice behaviour (Extended Data Fig. 2e).

For CsChrimson optogenetics experiments, flies were treated as above but were kept under low white light (about 3 nW mm^–2^ measured at 660 nm) from egg to adulthood. At around age 5–6 days, roughly 20 females were exposed to around 20 Canton-S males in an empty bottle containing only wet yeast paste and a Kimwipe soaked with 2 ml of 200 µM all-transretinal in water (also kept under low white light). Wet yeast paste was applied to the side of the bottle and comprised 1 g of dry yeast with 1.5 ml of 4.25 mM putrescine dihydrochloride and 200 µM all-transretinal in water. Flies were tethered about 24 h later. Flies for CsChrimson control experiments were always treated identically to CsChrimson-expressing flies.

Flies were anaesthetized at roughly 4 °C and tethered to a custom holder^67^, except where the back wall of the pyramid leading up to the fly was tilted at an angle rather than rising at 90°, to allow more light from the brain to reach the objective^66^ (Fig. 1d). The head was pitched forward during tethering to provide a view of oviDN cell bodies. For electrophysiology the head was inserted deeper into the holder for unobstructed access to oviDNs with electrodes. Flies were attached to the holder with blue-light-cured glue (Bondic). The proboscis was gently extended and the dorsal rostrum glued to the head capsule. This prevented brain movement associated with proboscis extension but still allowed measurement of proboscis extension (albeit with a smaller dynamic range than natural proboscis extension). Extracellular saline solution was added to the holder well (bath) and a window was cut in the cuticle with a 30-gauge needle (BD PrecisionGlide). The cuticle and some trachea were removed with forceps to expose the posterior aspect of the brain. The holder was stabilized with magnets above the egg-laying wheel inside a small custom humidification chamber.

Extracellular saline^68^ comprised 103 mM NaCl, 3 mM KCl, 5 mM *N*-Tris(hydroxymethyl) methyl-2-aminoethanesulfonic acid, 10 mM trehalose, 10 mM glucose, 2 mM sucrose, 26 mM NaHCO_3_, 1 mM NaH_2_PO_4_, 1.5 mM CaCl_2_ and 4 mM MgCl_2_. Osmolarity was 280 ± 5 mOsm and pH was 7.3–7.4 when bubbled with 95% O_2_/5% CO_2_. The temperature of the bath was set to around 17–22 °C by flowing fresh saline through a Peltier device with feedback from a thermistor in the bath (Warner Instruments).

### Calcium imaging

We used a two-photon microscope with a moveable objective (Ultima IV, Bruker) and custom stage (Thorlabs, Siskiyou). The microscope was controlled by Prairie View software (Bruker) and was enclosed by a black shroud. A Chameleon Ultra II Ti:Sapphire femtosecond pulse laser (Coherent) filtered by a 715 nm longpass filter (Semrock) provided 925 nm two-photon excitation. Emission light from the brain was collected by a ×16/0.80 numerical aperture (NA) objective (×16 W CFI75 LWD, Nikon), split by a 565 nm dichroic and filtered by a 490–560 nm bandpass filter (Chroma) before entering GaAsP detectors (Hamamatsu). For CsChrimson optogenetics experiments the emission light was split by a 525 nm dichroic and filtered by both a 490–510 nm and a 480–520 nm bandpass filter (Chroma) to prevent optogenetic stimulation light from entering the detector. A Piezo motor was used for volumetric scanning.

A range of optical zooms, z-slice number, z-slice separation, fields of view, laser powers (6–30 mW at the specimen) and frame rates (mean of 1.5 Hz) were used over the course of experiments on oviDN dynamics. Individual data traces were inspected by eye and the reported results were robust to the range of parameters used. All recordings had multiple z-slices within, above and below the cell body permitting effective quantification of recordings with slight z-drift over hours of recording. For example, in Fig. 1g, 14 z-slices were taken at 3 µm steps and only around five or six of these included fluorescence from the oviDNb cell body. The length of each recording (mean of 75 min) varied depending on (1) the perceived health of the fly, (2) the likelihood of future egg-laying events (which were higher if the fly had already laid an egg), (3) the amount of z-drift and (4) the quality of the agarose wheel, which sometimes visibly dried over a period of hours. The experimenter was blind to correlations between the neural signal and behaviour during the vast majority (roughly 95%) of recordings. Flies were excluded only if a technical issue arose (for example, errors in synchronizing behaviour with two-photon imaging or saline leaking from the holder). Only eggs with continuous two-photon imaging from 240 s before to 30 s after egg deposition were analysed.

For CsChrimson optogenetics experiments supporting a rise-to-threshold mechanism (Fig. 2f–h), two-photon imaging parameters were held relatively constant (mean frame rate of 1.5 Hz and two-photon laser power of approximately 10.5 mW). CsChrimson stimulation intensities were determined in pilot experiments. Periodic stimulation cycling four intensities was applied for 5 s every 2 min. The experimenter was blind to correlations between the neural signal and behaviour during all these recordings.

For CsChrimson manual stimulation experiments (Fig. 2a–c), stimulations were initiated by the experimenter while observing the real-time behaviour of the fly. Stimulations were initiated, on average, roughly once every 7.5 min. Manual stimulations were typically halted if the fly began to ovulate or it showed signs that it would ovulate soon (that is, pausing and slight abdominal elongation). Once ovulation was complete, stimulation was triggered when the fly’s abdomen was not touching the substrate (and before any indication that a spontaneous egg-laying event was about to take place). The traces shown in Fig. 2a (and associated Supplementary Video 5) are representative of our manual stimulation protocol. We used manual stimulation because it resulted in around a twofold higher rate of eggs laid than periodic stimulation, and also it allowed us to activate oviDNs after ovulation but before spontaneous egg laying.

For the CsChrimson optogenetics experiments shown in Fig. 2c, two-photon imaging data are shown for only five of the nine flies whereas behavioural data are shown for all nine. The four flies for which we do not show imaging data had bleed-through artefacts in the GCaMP signal from the CsChrimson illumination LED because these data were collected before optimization of the detection path for minimization of this artefact.

Two-photon imaging frames were motion corrected using either custom scripts from a previous study^66^ or CaImAn^69^. The regions of interest (ROIs) for a cell body were drawn manually for each *z*-plane using the time-average of each. ROIs were drawn around the outer boundary of the cell body. The brighter of the two cell bodies in oviDN-SS1 was assigned to be oviDNb (see Extended Data Fig. 3c, in which we show that the brighter of the two cells in oviDN-SS1 is oviDNb). In a few cases in which the brighter cell was not obvious, ROIs encompassing both cell bodies were drawn and assigned to be oviDNb. For a given imaging volume time point, the individual pixel intensities in all individual *z*-plane ROIs for a given cell were pooled and averaged, *F*_cell_(*t*). An identical average was calculated for a background volume of pixels that did not overlap the oviDN soma, or any other soma or neurite, *F*_background_(*t*). Before calculation of ∆*F*/*F* we subtracted the background from the cell, *F*_cell_actual_(*t*) = *F*_cell_(*t*) – *F*_background_(*t*). This eliminated non-cell-specific signal such as autofluorescence and constant detector background. This subtraction also made ∆*F*/*F* robust to variations in the number of background pixels included in ROIs drawn around the outside of a cell. ∆*F*/*F* was calculated using the formula (*F*_cell_actual_(*t*) – *F*_0_(*t*))/*F*_0_(*t*), where *F*_0_(*t*) is the running mean of *F*_cell_actual_(*t*) over a 20 min window. The mean over a long time frame was used to estimate a baseline, systematically, for the continuously fluctuating oviDN signal. A similar running mean baseline estimate (albeit with a much shorter window) was previously used to quantify continuously fluctuating dopaminergic signals in mammals^70^. A ∆*F*/*F* of 0.35, for example, indicated that the fluorescence signal in the cell was 35% greater than the 20-min-mean signal in the cell. If the GCaMP7f fluorescence signal is linear with [Ca^2+^] in this range it would indicate that [Ca^2+^] in the cell had increased by 35% over the 20-min-mean [Ca^2+^] in the cell. All stated conclusions were robust to three different methodologies for calculation of ∆*F*/*F*, including methods where *F*_0_ remained constant. For CsChrimson experiments, *F*_0_(*t*) was the running mean of *F*_cell_actual_(*t*) over a 20 min window after the 105 s post-triggering CsChrimson stimulation had been set to NaN. This very conservatively prevented any CsChrimson stimulations, or lingering effects, from artificial influence of *F*_0_(*t*). Note that, because both baseline spike rate and *V*_m_ are higher for flies expressing CsChrimson (Extended Data Fig. 6a,b; approximately 12 spikes s^–1^ and –44 mV) than for those that are not (Extended Data Fig. 9a; approximately four spikes s^–1^ and –57 mV), we would expect the mean GCaMP signal that we use for normalization in CsChrimson flies to be reflective of higher calcium concentrations, resulting in lower ∆*F*/*F* values for the same absolute calcium concentration. For this reason—and because our genetic driver in CsChrimson experiments is expressed in only two of three oviDNs per side—quantitative comparisons of ∆*F*/*F* in CsChrimson and non-CsChrimson flies are not warranted.

Two-photon imaging-frame pulses, behavioural camera frame triggers and optogenetic LED triggers were all digitized at 10 kHz on a Digidata 1440A (Molecular Devices) and saved to a computer (Axoscope, Molecular Devices). To assign a timestamp to a volume scan we identified the moment that the two-photon volume scan was half complete. To assign a timestamp to a behavioural camera frame we used the beginning of the 16 ms camera exposure period. Calcium imaging was interpolated and behavioural data were downsampled to a common 10 Hz array for all population analyses. Each 100 ms time point was assigned the calcium imaging and behaviour data value from the closest previous respective timestamp (that is, previous neighbour interpolation). A relatively large 100 ms time base was chosen because faster sampling was unnecessary for the current analyses and would be computationally time consuming given the 200+ h of two-photon scanning collected. In the case of triggered averages, the zero point was either the timestamp for the behaviour camera frame with the behaviour of interest or the frame with the onset of optogenetic stimulation. In the case of cross-correlations, the zero point was the timestamp of the first acquired two-photon volume.

### Electrophysiology

We used the same two-photon microscope for both calcium imaging and patch-clamp electrophysiology. The microscope was controlled by either Prairie View (Bruker) or µManager^71^ software. A 470 nm LED (pE-100, CoolLED) provided excitation through the objective to identify 2× EGFP- or GCaMP7f-positive neurons. An 850 nm LED coupled to a 400-µm-wide fibre-optic cable (M850F2 and M28L01, Thorlabs) was focused on the fly’s head to illuminate cells for patch-clamping using a lens set (MAP10100100-A, Thorlabs). Both LEDs were turned off when recording electrophysiology data. A ×40/0.80 NA objective (LUMPLFLN 40XW, Olympus) and CoolSnapEZ CCD camera (Photometrics) were used for patch-clamping.

Cell bodies were exposed by breaching the neural lamella and perineural sheath using gentle application of 0.5% collagenase IV (Worthington) in extracellular saline via pipette. We applied collagenase IV to a small 30 × 30 µm^2^ area containing the cell bodies of interest^67^. Collagenase was applied using a 4–6-µm-tip micropipette with 8–80 mmHg positive pressure at around 30–32 °C for about 3 min. Once the cell bodies were exposed, the bath was returned to about 19–21 °C and flushed free of collagenase.

Borosilicate glass (outer diameter 1.5 mm, inner diameter 0.86 mm, with filament) was pulled to create a 7–15 MΩ electrode with a 1.0–1.5 µm tip using a model P-1000 micropipette puller (Sutter Instruments) and fire-polished with a MF-900 Microforge (Narishige). Intracellular saline^68^ comprised 140 mM potassium-aspartate, 1 mM KCl, 10 mM HEPES, 1 mM EGTA, 0.5 mM Na_3_GTP, 4 mM MgATP, 13 mM biocytin hydrazide and 20 µM Alexa-568–hydrazide-Na (ThermoFisher Scientific). The pH was adjusted to about 7.3 with KOH, and osmolarity to approximately 265 mOsm with water.

Electrophysiological signals were acquired using a MultiClamp 700B amplifier (Molecular Devices) in current-clamp mode. Electrophysiological signals and behavioural camera triggers were digitized at 10 kHz via a Digidata 1440A and saved to a computer (Clampex, Molecular Devices). The oviDN or oviDN-like subtype (Extended Data Fig. 3) from which recording was taken was not distinguished. Electrophysiology experiments using oviDN-SS1 could target oviDNa or oviDNb and experiments using oviDN-GAL4 could target oviDNa, oviDNb or oviDN-like neurons. Recordings were made without current injection (except for current step protocols) and the reported membrane voltage (*V*_m_) was corrected for a 13 mV junction potential^67^. Spikes were identified by highpass filtering *V*_m_ and finding peaks above a threshold that were separated in time by over 1 ms. Parameters for peak detection were varied from recording to recording based on visual inspection of the data, in which the action potentials were clear. We calculated the spike rate by counting the number of spikes in every 5 s interval (at 0.1 ms steps), dividing by 5 and assigning that value to the middle of the 5 s interval (for Extended Data Fig. 6b,e a 100 ms rather than 5 s interval was used). Spike rate and *V*_m_ were thus both measured at 0.1 ms intervals. Data were aligned and analysed identically to calcium imaging. Resting *V*_m_ was considered the first stable *V*_m_ after breaking into the whole-cell configuration (Fig. 5d and Extended Data Fig. 9a). We calculated a *V*_m_ with spikes removed by discarding (converting to NaNs) 150 ms of data centred on the peak of each spike (Extended Data Fig. 9c).

Electrophysiological recordings for 2× EGFP-expressing flies were analysed only if (1) the cell was stably recorded for more than 3 min; (2) *V*_m_ was below –43 mV at rest with no large drift or rapid fluctuations that were clearly non-physiological; (3) the fly walked for at least one wheel rotation; and (4) the cell spiked at least once. A total of five cells were rejected for not passing criteria 2, 3 and 4. Three of these five were rejected for not passing criterion 2, and a single cell was rejected for not passing criterion 3, indicating that flies were healthy in this preparation. A single cell passed the first three criteria but was rejected for not spiking (shown in Extended Data Fig. 9a). Cells that passed all four criteria were analysed from the time when the recording first stabilized to when it degraded or was terminated (mean, 41 min).

Electrophysiological recordings for CsChrimson-expressing flies were analysed if *V*_m_ was below –43 mV at rest. All recordings were conducted in vivo and with the fly on the wheel.

Electrophysiological recordings for Kir2.1*- and Kir2.1*Mut-expressing flies were analysed if *V*_m_ was below –43 mV at rest. These flies were pretreated as described for free-behaviour experiments rather than as described for tethered experiments, so the transgene would be expressed because it was in free behaviour. All recordings were done in vivo on the wheel. Current step protocols were conducted with 5 pA increments with 1 s of current injection (Extended Data Fig. 10d).

### Abdominal ganglion calcium imaging

Flies were anaesthetized at approximately 4 °C and their wings clipped near their base before tethering to a custom holder. The holder was similar to that used in our other experiments except that it lacked a pyramid (such that the objective could be lowered to image deep ventral tissue) and had a larger hole (such that the head, thorax and anterior-most part of the abdomen could fit, rather than just the head) (Extended Data Fig. 6h–j). The dorsal part of the thorax was pushed through the hole and the posterior head was aligned and pitched in the hole to be in plane with the holder. The thorax, abdomen and head were glued to the holder with blue-light-cured glue (Bondic). Glue was applied to the anterior abdomen to stabilize the preparation and avoid tearing of the delicate cuticle of the abdomen during dissection. As a result, the fly was not able to bend its abdomen normally. The rostrum was not glued in this preparation because proboscis extension did not cause movements in the abdominal ganglion as it did in the brain. A needle was used to slice a window in the cuticle of the dorsal thorax (Extended Data Fig. 6j; blue box shows dissection area), and the cuticle and indirect flight muscles were removed with forceps such that the dorsal proventriculus and surrounding trachea were visible. Removal of the indirect flight muscles was easier without extracellular saline solution in the bath and thus was done quickly (within 30 s) to prevent desiccation. Extracellular saline was then added. The section of the proventriculus near the neck connective was cut, and the portions of the gut covering the ventral nerve cord, as well as the trachea and crop, were removed. The preparation was flushed with extracellular saline to dilute digestive enzymes that might have been released during dissection. Loose tissue (for example, remaining indirect flight muscles) was carefully removed or retracted such that the abdominal ganglion was visible. Despite removal of several dorsal structures to expose the ventral nerve cord and abdominal ganglion, flies were able to walk. Occasional flies that were not able to move their legs normally either before or after imaging were discarded. Overall, tethering and dissection shared features with previous work^72^ except that significant time and effort were needed to advance dissection past the neck connective, T1, T2 and T3 neuromeres to the abdominal ganglion. (Previous imaging in walking flies was restricted to the more accessible neck connective and T1 neuromere^72,73^). The holder was stabilized with magnets above the egg-laying wheel inside a small custom humidification chamber.

Calcium imaging rates of around 0.5 Hz and laser powers of 20–30 mW at the specimen were needed to capture sufficient signal and sample the full presynaptic volume (approximately 50 × 50 × 60 µm^3^). Imaging rates and laser powers are similar in Extended Data Fig. 6l,m, to aid comparison (mean imaging rate of 0.50 and 0.56 Hz, respectively, with a minimum rate of all data at 0.36 Hz). Because the timestamp assigned to a volume scan was when the volume was half complete, data of around 1 s after cessation of stimulation in Extended Data Fig. 6l,m should minimally include the stimulation period; data 1.4 s (delay for 0.36 Hz) after stimulation do not include the stimulation period at all. These numbers also apply to the increase in ∆*F*/*F* at stimulation onset. Half-decay times are the amount of time after cessation of stimulation required for signal value to return half-way between that at the end of stimulation and 5 s mean prestimulation. The half-decay times reported in the main text are the average of those for the three lower intensities shown in Extended Data Fig. 6d–f. To calculate an expected ∆*F*/*F* half-decay time given a spike rate, GCaMP7f kinetics and calcium imaging rate, we convolved one of our spike-rate traces (second-lowest intensity shown in Extended Data Fig. 6e) with an exponential filter (*τ* = 300 ms) that estimates the off kinetics of GCaMP7f^17^ and then applied a boxcar filter (width, 2.8 s) that simulated the slowest frame rate in all experiments. The half-decay time of this simulated trace was 700 ms.

### Substrate transition-triggered averages during calcium imaging or electrophysiology

Substrate transitions were identified using the fly’s position on the wheel. For these analyses, substrate transition *i* was eliminated if substrate transitions *i* – 1 and *i* + 1 occurred within 4 s of each other. This empirically prevented events in which the fly rocked on the substrate boundary from being counted as multiple transitions. Note that, for all transition-triggered averages, if the fly were to have transitioned back to the original substrate—say, 20 s after the first transition—the data from 20 s onwards would not contribute to the post-transition average.

### Measurement of light power

All light power levels reported in this paper were measured with a PM100D Compact Power and Energy Console (Thorlabs) at the expected peak intensity of the light source. Lighting with an area smaller than the sensor was divided by the estimated illuminated area rather than by the area of the sensor.

### Texas Red fill

Texas Red (100 mg ml^–1^; dextran, Texas Red, 3,000 MW, lysine fixable) (ThermoFisher Scientific) in patch-clamp intracellular saline (see above) lacking ATP, GTP, biocytin and Alexa-568–hydrazide-Na was backfilled into a patch pipette. The pipette was positioned near the cell body (without any collagenase application) and two to five pulses of 10 V (2 ms duration) were applied using an SD9 stimulator (Grass Instruments). All fills and anatomy were carried out with flies on the wheel under the two-photon microscope (as in calcium imaging, except using a ×40/0.80 NA objective (LUMPLFLN 40XW, Olympus) and a 590–650 nm bandpass filter (Chroma) to filter emitted light before entering a second GaAsP detector (Hamamatsu).

### Split-GAL4 screening and stabilization

Split-GAL4 lines were screened and stabilized as described previously^9^. To determine cell types labelled by a particular split-GAL4 driver, standard immunofluorescence staining was used to count the total number of cells (Extended Data Fig. 11a–r) and stochastic labelling in multiple colours^74^ was used to visualize the morphology of individual cells. Individual cell morphology was used to manually assign cells to hemibrain connectome body IDs^16^, and the cell type and instance associated with the body ID were noted (Supplementary Table 3).

In Fig. 6a the number of pairs of oviDN input neurons is an estimate based on correspondence between light microscopy images of neurons labelled in split-GAL4 and the hemibrain connectome^16^ (above); the number of synapses onto oviDNs is an estimate of the total number of synapses onto oviDNs from those neurons using the electron microscopy connectome (Supplementary Table 3).

### Analysis of hemibrain for recurrent-circuit inputs to oviDNs

We analysed synaptic connections in the adult female hemibrain using the neuPrint^75^ (v.1.2.1) Python interface. All connections with at least one synapse per connection were queried for the circuit architectures investigated (Extended Data Fig. 11s–v). Because oviDNs receive an enormous number (approximately 600–1,100) of input synapses and have very few (roughly between five and 50) output synapses in the hemibrain, direct, two-way reciprocal connections between pairs of oviDNs—or between oviDNs and other cells—were not evident (Extended Data Fig. 11s,t, also diagrammed in Fig. 6d). Of all the recurrent circuits (with at least ten synapses) in the hemibrain that directly involve oviENs—which are the dominant input cells to oviDNs—the neurons diagrammed in Fig. 6e are the only ones that concisely/directly interconnect oviENs on both sides specifically via a single group G, group U or oviIN cell. We could not discover a recurrent circuit that uses a single cell class to interconnect oviDNs on both sides using the same ten-synapse threshold (Extended Data Fig. 11u,v). (Interconnection of oviDNs on both sides is a sensible constraint for an underlying circuit because the calcium signals of oviDNs on both sides track tightly during egg laying.) Although we did not find simpler recurrent-circuit architectures (Extended Data Fig. 11s–v), complementary circuits could still exist particularly in regions of the nervous system where connectome data are unavailable, or via gap junctions, which are not annotated in existing fly connectomes.

Although inhibition of group Z neurons also had an effect on eggs laid (Fig. 6a), 11 of 18 flies with inhibition of group Z still laid more than one egg. Note that group Z neurons provide synaptic input to oviDNs, oviENs, group G and group U cells (Supplementary Table 4), potentially explaining why flies lay fewer eggs when these neurons are inhibited (Fig. 6a). Group Z neurons, however, receive few synapses back from the other relevant cell classes and they thus reside, in our interpretation, outside of the core loop.

The fact that recurrent-circuit neurons on both sides of the brain are reciprocally connected helps to explain why the oviDN [Ca^2+^] signal on both sides is qualitatively similar (Extended Data Fig. 3f). Group U cells and at least one group G cell were positive for tyrosine hydroxylase (Extended Data Fig. 12), suggesting that the physiology of this recurrent circuit may be more sophisticated than one in which all circuit elements express the same excitatory transmitter to implement simple, runaway excitation^76,77^.

### Immunofluorescence staining and confocal microscopy

Immunofluorescence staining and confocal microscopy were performed as described previously^9^ (with modifications for Extended Data Fig. 12; see below), using the following antibodies and dilutions: 1:30 mouse anti-Bruchpilot (no. nc82, Developmental Studies Hybridoma Bank), 1:300 rabbit anti-HA Tag (no. 3724S, Cell Signaling Technology), 1:200 rat anti-FLAG Tag (no. NBP1-06712, Novus Biologicals), 1:500 DyLight 550 mouse anti-V5 Tag (no. MCA1360D550GA, AbD Serotec), 1:500 Alexa Fluor 594 donkey anti-rabbit (no. 711-585-152, Jackson ImmunoResearch), 1:600 ATTO 647N goat anti-rat (no. 612-156-120, Rockland), 1:600 Cy2 goat anti-mouse (no. 115-225-166, Jackson ImmunoResearch), 1:800 Alex Flour 488 goat anti-rabbit (no. A11034, Thermo Fisher Scientific), 1:400 AlexaFlour568 goat anti-mouse (no. A11031, Thermo Fisher Scientific) and 1:1,000 rabbit anti-GFP (no. A11122, Thermo Fisher Scientific).

For identification of neurotransmitter identity (Extended Data Fig. 12), brains were dissected in cold Schneider’s insect medium (no. S0146, Sigma-Aldrich) and fixed overnight at 4 °C in Schneider’s insect medium with 1% paraformaldehyde (no. 15713, Electron Microscopy Science). For vGluT staining, brains were instead fixed for 5 min at room temperature in Bouin’s fixative (no. 112016, Ricca Chemical Co.) as described previously^78^. Primary antibodies and their dilutions were: 1:300 rabbit anti-TH (no. AB152, Sigma-Aldrich), 1:500 rabbit anti-serotonin (no. S5545, Sigma-Aldrich), 1:50 mouse anti-ChAT (no. ChAT4B1-s, Developmental Studies Hybridoma Bank), 1:500 rabbit anti-GABA (no. A2052, Sigma-Aldrich), 1:10,000 rabbit anti-vGluT^78^ (gift from A. DiAntonio), 1:1,000 chicken anti-GFP (no. 600-901-215, Rockland) and 1:30 mouse anti-Bruchpilot (no. nc82, Developmental Studies Hybridoma Bank) for all but anti-ChAT experiments. Secondary antibodies and their dilutions were: 1:800 goat anti-chicken Alexa Flour 488 (no. A11039, Thermo Fisher Scientific), 1:400 goat anti-mouse Alexa Flour 594 (no. A11032, ThermoF isher Scientific) and 1:400 goat anti-rabbit Alexa Flour 633 (no. A21070, Thermo Fisher Scientific). Samples were mounted in Vectashield H-1000 (Vector Laboratories) and imaged on an LSM 780 confocal microscope (Zeiss) with a ×20/0.8 NA objective (no. 440640-9903-000, Zeiss) at 1 µm z-intervals. Images were analysed using Fiji (ImageJ).

### Statistics and reproducibility

We used the two-sided Wilcoxon rank-sum test to calculate all *P* values.

For egg-laying choice fractions (for example, Fig. 3b), grey bars indicate the fraction of eggs laid on the lower-sucrose option after all eggs from all flies are pooled. Error bars indicate the 95% confidence interval of this fraction calculated using the Clopper–Pearson method (‘exact’ binomial confidence interval). Individual dots represent individual flies.

The first two *P* values in the main text compare the number of trials with (or without) events in two separate groups. For a single group, trials with an event are treated as 1 and those without an event are treated as 0. The two groups (each a set of 0 and 1) are then compared using the two-sided Wilcoxon rank-sum test (*P* values calculated using two-sided Fisher’s exact test are similar and similarly significant). Exact *P* values in the main text are 2.1 × 10^–25^, 8.4 × 10^–29^ and 3.3 × 10^–54^.

For the calculations shown in Fig. 4f we used the point at which ∆*F*/*F* crosses 0 as the starting point in the slope calculation, because it relies solely on the ∆*F*/*F* signal and not behaviour. A ∆*F*/*F* value of 0 is, on average, related to the beginning of the search (Fig. 1k). *P* values were calculated using the two-sided Wilcoxon rank-sum test; *P* = 0.030 when comparing slopes in the 25 s before abdomen bend and *P* = 0.064 when comparing slopes from search start to abdomen bend.

Immunofluorescence examples shown in Fig. 1f and Extended Data Figs. 3b, 11a–r and 12 are representative of at least two brains (four total brain sides) and typically more than three brains (six total brain sides). Electron microscopy-based anatomy shown in Fig. 6c and Extended Data Fig. 3a was generated from a single side of one brain^16^.

No data were excluded unless explicitly stated. No statistical method was used to choose sample size. Experimenters were not blind to fly genotype. Flies were randomly chosen for each experiment.

### Data analysis

All data analyses and instrument control were done using either MATLAB (MathWorks) or Python unless otherwise specified. All design for 3D printing or laser cutting was done using Autodesk Inventor (Autodesk), which was also used to help create Fig. 1d and Extended Data Fig. 2a–c.

### Reporting summary

Further information on research design is available in the Nature Portfolio Reporting Summary linked to this article.

## Online content

Any methods, additional references, Nature Portfolio reporting summaries, source data, extended data, supplementary information, acknowledgements, peer review information; details of author contributions and competing interests; and statements of data and code availability are available at 10.1038/s41586-023-06271-6.

## Supplementary information

Supplementary Discussion

Reporting Summary

Supplementary Table 1 Genotypes for each experiment. All flies used for experiments were female.

Supplementary Table 2 Conditions for each experiment.

Supplementary Table 3 Characterization of split-GAL4 lines for oviDN input neurons. Numbers in table are synapse counts from row (presynaptic) to column (postsynaptic) using the hemibrain connectome^16^.

Supplementary Table 4 Connectivity of oviDN input neurons that reduce the total number of eggs laid when silenced. Numbers in table are synapse counts from row (presynaptic) to column (postsynaptic) using the hemibrain connectome^16^.

Supplementary Video 1 Individual egg-laying event of a fly expressing GCaMP3 in eggs. An egg-laying event with the behavioural sequence occurring in a localized space was chosen so that it could be magnified. Video is compressed and played back at 5× speed.

Supplementary Video 2 Three consecutive egg-laying events of a wild-type fly in a sloped-ceiling egg-laying chamber (as in Extended Data Fig. 1d). Traces below video are smoothed with a 5 s boxcar filter. Pink line is overlaid on the fly to indicate neck to ovipositor length and is drawn only in frames that passed the criteria described in Methods. Video is compressed and played back at 1× speed.

Supplementary Video 3 Egg-laying event during two-photon imaging in a tethered fly expressing GCaMP7f in oviDNs (as in Fig. 1g, left). ∆*F*/*F* and brain images are smoothed with a 2 s boxcar filter. Video is compressed and played back at 5× speed.

Supplementary Video 4 Egg-laying event during two-photon imaging in a tethered fly expressing GCaMP7f in oviDNs (as in Fig. 1g, right). ∆*F*/*F* and brain images are smoothed with a 2 s boxcar filter. Video is compressed and played back at 5× speed.

Supplementary Video 5 Optogenetically stimulated egg-laying event and abdomen bends in a tethered fly expressing GCaMP7f and CsChrimson in oviDNs (as in Fig. 2a, left). CsChrimson stimulations here were manually triggered. An orange dot in close-up of fly and orange line in ‘fly position’ trace both indicate stimulation periods. ∆*F*/*F* and brain images are smoothed with a 2 s boxcar filter. Video is compressed and played back at 5× speed.

Supplementary Video 6 Wild-type flies in a high-throughput 0 (bottom) versus 500 (top) mM egg-laying chamber. Video is compressed and accelerated ×60 by displaying only every 60th frame. Videos analysed for figures included all frames and were therefore much smoother.

## Data Availability

All calcium imaging and fly behaviour time-course datasets analysed in the main figures are available on DANDI archive (calcium imaging data, 000247; fly choice tracking data, 000212; fly behavioural sequence tracking data, 000250). Technical documents (for example, CAD files and plasmid maps) and source data for all scatter plots and histograms are available on Figshare (10.6084/m9.figshare.c.6505732).

## Code availability

Scripts for data processing and plotting are available on request.
