# Supplementary material for: A rise-to-threshold process for a relative-value decision
Source: Nature. 2023 Jul 5;619(7970):563–71. doi: 10.1038/s41586-023-06271-6 (PMC10356611; doi:10.1038/s41586-023-06271-6)
Supplement: Supplementary file 1 — Supplementary Discussion [file 41586_2023_6271_MOESM1_ESM.pdf]

nature portfolio

https://doi.org/10.1038/s41586-023-06271-6

Supplementary information

A rise-to-threshold process for a relative-value decision

In the format provided by the authors and unedited

Supplementary Information

Supplementary Discussion

Calcium dynamics in the oviDN axon terminals.

We tested whether the synaptic terminals of oviDNs, which are located in the abdominal ganglion, express similar [Ca^{2+}] dynamics to those observed in the oviDN soma. We imaged synaptically targeted GCaMP7f [80,81] in the axon terminals of oviDNs during CsChrimson stimulation as flies walked on our egg-laying wheel (Methods). Note that sytGCaMP biases GCaMP expression to terminals, but not necessarily to active zones[81]. When averaging the sytGCaMP signal across a large field of oviDN terminals, we observed slow [Ca^{2+}] dynamics in response to CsChrimson-mediated conductance changes, with a ΔF/F half-decay time of ~2.5 s across the population (Extended Data Fig. 6h-l) (Methods). A 2.5 s decay time is faster than the ~5.0 s decay time we measured at the soma in parallel experiments (Extended Data Fig. 6m), but still longer than the ~700 ms expected if [Ca^{2+}] were to tightly follow the spike rate with only the off kinetics of GCaMP slowing the signal (Methods). We also measured GCaMP in individual axonal boutons. For these measurements, to get a stronger signal, we used standard, non-synaptically targeted, GCaMP7f and analyzed ROIs around individual synaptic terminals. Some individual terminals—perhaps the functionally relevant ones—expressed decay times similar to or exceeding the 5.0 s observed in the soma (Extended Data Fig. 6n-p). While it is not yet possible to image [Ca^{2+}] in the abdominal ganglion during spontaneous egg laying—and [Ca^{2+}] signals in the terminals are themselves only a proxy for the complex biochemistry underlying synaptic transmission—these results support the interpretation that the [Ca^{2+}] signal we measure in the soma has similarities to [Ca^{2+}] signals in the cell's terminal regions, where we posit the threshold may be ultimately be implemented.
