# Supplementary material for: A rise-to-threshold process for a relative-value decision
Source: Nature. 2023 Jul 5;619(7970):563–71. doi: 10.1038/s41586-023-06271-6 (PMC10356611; doi:10.1038/s41586-023-06271-6)
Supplement: Supplementary file 2 — Reporting Summary [file 41586_2023_6271_MOESM2_ESM.pdf]

nature portfolio

Corresponding author(s): Gaby Maimon, Vikram Vijayan

Last updated by author(s): 4/20/2023

Reporting Summary

Nature Portfolio wishes to improve the reproducibility of the work that we publish. This form provides structure for consistency and transparency in reporting. For further information on Nature Portfolio policies, see our Editorial Policies and the Editorial Policy Checklist.

Statistics

For all statistical analyses, confirm that the following items are present in the figure legend, table legend, main text, or Methods section.

n/a	Confirmed	
☐	☒	The exact sample size (n) for each experimental group/condition, given as a discrete number and unit of measurement
☐	☒	A statement on whether measurements were taken from distinct samples or whether the same sample was measured repeatedly
☐	☒	The statistical test(s) used AND whether they are one- or two-sided<br>*Only common tests should be described solely by name; describe more complex techniques in the Methods section.*
☐	☒	A description of all covariates tested
☐	☒	A description of any assumptions or corrections, such as tests of normality and adjustment for multiple comparisons
☐	☒	A full description of the statistical parameters including central tendency (e.g. means) or other basic estimates (e.g. regression coefficient) AND variation (e.g. standard deviation) or associated estimates of uncertainty (e.g. confidence intervals)
☐	☒	For null hypothesis testing, the test statistic (e.g. F, t, r) with confidence intervals, effect sizes, degrees of freedom and P value noted<br>*Give P values as exact values whenever suitable.*
☒	☐	For Bayesian analysis, information on the choice of priors and Markov chain Monte Carlo settings
☒	☐	For hierarchical and complex designs, identification of the appropriate level for tests and full reporting of outcomes
☐	☒	Estimates of effect sizes (e.g. Cohen's d, Pearson's r), indicating how they were calculated

Our web collection on statistics for biologists contains articles on many of the points above.

Software and code

Policy information about availability of computer code

Data collection	Two-photon images were collected using PrairieView 5.4 software (Bruker), and associated triggers were collected using Axoscope 10.5.1.0 (Molecular Devices). Two-photon motion correction was done using custom scripts or CaImAn package (Flatiron Institute). Electrophysiology data and associated triggers were collected using Clampex 10.5.1.0 software (Molecular Devices), and the microscope was controlled using either PrarieView 5.4 software (Bruker) or uManager 1.4 software. Light pulses for optogenetics were controlled using custom Matlab code (Mathworks). Animals were monitored using FLIR cameras and FlyCapture 2.13.3.61 software (FLIR) or Hamamatsu cameras and HCImage 4.5.1.3 software (Hamamatsu). Flies and egg-laying wheel were tracked using Ctrax 0.5 software and/or Deeplabcut 2.0 software.
Data analysis	Electron microscopy connectome data was analyzed using Neuprint Python interface (Python 3.8). Confocal images were analyzed using Fiji (ImageJ version 1.53). Data were analyzed using custom Matlab scripts (Mathworks, Matlab 2021a). Scripts are available from the corresponding authors on request.

For manuscripts utilizing custom algorithms or software that are central to the research but not yet described in published literature, software must be made available to editors and reviewers. We strongly encourage code deposition in a community repository (e.g. GitHub). See the Nature Portfolio guidelines for submitting code & software for further information.

Data

Policy information about availability of data

All manuscripts must include a data availability statement. This statement should provide the following information, where applicable:

- Accession codes, unique identifiers, or web links for publicly available datasets
- A description of any restrictions on data availability
- For clinical datasets or third party data, please ensure that the statement adheres to our policy

All calcium imaging and fly behavior time course datasets analyzed in the main figures are available on DANDI archive (calcium imaging data: 000247, fly choice tracking data: 000212, fly behavioral sequence tracking data: 000250). Technical documents (e.g., CAD files and plasmid maps) and source data for all scatter plots and histograms are available on Figshare (https://doi.org/10.6084/m9.figshare.c.6505732). Scripts for data processing and plotting are available upon request.

Human research participants

Policy information about studies involving human research participants and Sex and Gender in Research.

Reporting on sex and gender	No human research participants in this work.
Population characteristics	No human research participants in this work.
Recruitment	No human research participants in this work.
Ethics oversight	No human research participants in this work.

Note that full information on the approval of the study protocol must also be provided in the manuscript.

Field-specific reporting

Please select the one below that is the best fit for your research. If you are not sure, read the appropriate sections before making your selection.

☒ Life sciences ☐ Behavioural & social sciences ☐ Ecological, evolutionary & environmental sciences

For a reference copy of the document with all sections, see nature.com/documents/nr-reporting-summary-flat.pdf

Life sciences study design

All studies must disclose on these points even when the disclosure is negative.

Sample size	No statistical tests were used to determine sample size. Samples sizes were determined by expected fly-to-fly variability from our pilot experiments and from experiments conducted by others in the field. Our sample sizes (# of flies assayed) are in line with those used in similar experiments in previous studies. For behavioral experiments see: Wang et al Nature 2022, Yang et al Journal of Neuroscience 2015. For physiology during behavior experiments see: Seelig & Jayaraman Nature 2015, Green et al. Nature 2017.
Data exclusions	No flies were excluded for free behavior experiments. Flies were only excluded for two-photon imaging experiments if a technical issue arose (e.g., errors synchronizing behavior with two-photon imaging or saline leaking from the holder) (see Methods for details). Flies were only excluded for electrophysiology experiments if they were not healthy enough to complete a single wheel rotation or the recording was unstable (see Methods for details). Criteria for data exclusions were established after pilot experiments.
Replication	All attempts at replication were successful and preliminary experiments were consistent with experiments reported in manuscript. Experiments were almost always done on multiple days, and day specific effects were not observed. Experiments were almost always done with multiple fly crosses (i.e. to rule out fly handling issues), and fly cross specific effects were not observed. The data in this manuscript combines data from all replicates (no data excluded, unless as noted). In cases where example confocal images are shown (Fig. 1f, Extended Data Fig. 3b, Extended Data Fig. 11a-r, and Extended Data Fig. 12), the examples are representative of at least 4 brain sides and typically more than 6 brain sides.
Randomization	Flies were randomly chosen for each experiment. Samples were allocated based on the corresponding genotypes.
Blinding	During manual behavioral annotations, the experimenters were blind to any neuronal signals recorded (see Methods for details). During counting of eggs, the experimenters were not blind to the flies' genotypes as these counts were not subjective. In all other cases, data collection and analysis were done computationally, and thus the experimenters were not blind to the flies' genotypes.

Reporting for specific materials, systems and methods

We require information from authors about some types of materials, experimental systems and methods used in many studies. Here, indicate whether each material, system or method listed is relevant to your study. If you are not sure if a list item applies to your research, read the appropriate section before selecting a response.

Materials & experimental systems

n/a	Involved in the study
☐	☒ Antibodies
☒	☐ Eukaryotic cell lines
☒	☐ Palaeontology and archaeology
☐	☒ Animals and other organisms
☒	☐ Clinical data
☒	☐ Dual use research of concern

Methods

n/a	Involved in the study
☒	☐ ChIP-seq
☒	☐ Flow cytometry
☒	☐ MRI-based neuroimaging

Antibodies

Antibodies used	1:30 mouse anti-Bruchpilot (nc82, Developmental Studies Hybridoma Bank), 1:300 rabbit anti-HA Tag (3724S, Cell Signaling Technology), 1:200 rat anti-FLAG Tag (NBP1-06712, Novus Biologicals), 1:500 DyLight 550 mouse anti-V5 Tag (MCA1360D550GA, AbD Serotec), 1:500 Alexa Fluor 594 donkey anti-rabbit (711-585-152, Jackson ImmunoResearch) , 1:600 ATTO 647N goat anti-rat (612-156-120, Rockland), 1:600 Cy2 goat anti-mouse (115-225-166, Jackson ImmunoResearch), 1:800 Alex Flour 488 goat anti-rabbit (A11034, ThermoFisher Scientific), 1:400 AlexaFlour568 goat anti-mouse (A11031, ThermoFisher Scientific), 1:1000 rabbit anti-GFP (A11122, ThermoFisher Scientific), 1:300 rabbit anti-TH (AB152, Sigma-Aldrich), 1:500 rabbit anti-serotonin (S5545, Sigma-Aldrich), 1:50 mouse anti-ChAT (ChAT4B1-s, Developmental Studies Hybridoma Bank), 1:500 rabbit anti-GABA (A2052, Sigma-Aldrich), 1:10,000 rabbit anti-vGluT (gift from Aaron DiAntonio, Daniels et al 2004); 1:1,000 chicken anti-GFP (600-901-215, Rockland), 1:800 goat anti-chicken Alexa Flour 488 (A11039, ThermoFisher Scientific), 1:400 goat anti-mouse Alexa Flour 594 (A11032, ThermoFisher Scientific), and 1:400 goat anti-rabbit Alexa Flour 633 (A21070, ThermoFisher Scientific).
Validation	All antibodies are part of extensively used Drosophila protocols including protocols established by the Janelia FlyLight team (https://www.janelia.org/project-team/flylight/protocols). Additionally, primary antibodies used in this study were extensively validated for use with immunohistochemistry and Drosophila species as described on the company websites and references therein (see catalog number and company above). Validation of the vGluT antibody was done in Daniels et al 2004, and the antibody has been extensively used in many Drosophila studies since.

Animals and other research organisms

Policy information about studies involving animals; ARRIVE guidelines recommended for reporting animal research, and Sex and Gender in Research

Laboratory animals	3 to 7 day old Drosophila melanogaster (invertebrate species) females were used in this study. Detailed information concerning fly strains and their genotype are provided in the Supplement.
Wild animals	The study did not involve wild animals.
Reporting on sex	This study only used female Drosophila because it investigates the behavioral and neuronal mechanisms of egg laying -- a female specific behavior.
Field-collected samples	The study did not involve samples collected from the field.
Ethics oversight	No ethical approval is required for work in Drosophila melanogaster.

Note that full information on the approval of the study protocol must also be provided in the manuscript.
